# GFP chromophore photophysics: ultrafast dynamics and hot ground state cooling in the neutral form

**DOI:** 10.1039/d6sc02114j

**Published:** 2026-06-22

**Authors:** Anam Fatima, Mark H. Stockett, Eleanor K. Ashworth, Woojin Park, Cheol Ho Choi, Joseph A. Wright, Pratip Chakraborty, Partha Malakar, Stephen R. Meech, James N. Bull

**Affiliations:** a Chemistry, Faculty of Science, University of East Anglia Norwich NR4 7TJ UK; b Department of Physics, Stockholm University SE-10691 Stockholm Sweden; c Department of Chemistry, Kyungpook National University Daegu 41566 South Korea; d Central Laser Facility, Research Complex at Harwell, Rutherford Appleton Laboratory Didcot OX11 0QX UK james.bull@uea.ac.uk

## Abstract

The neutral GFP chromophore is the photoactive form in many photoconvertible and photoswitchable fluorescent proteins, yet experimental characterisation of its ultrafast dynamics requires clear assignment of the associated transient signals. Defining these dynamics is important for understanding how protonation state tunes the intrinsic photophysics of GFP chromophores. Using complementary ultrafast electronic and vibrational spectroscopies supported by explicit-solvent calculations and spectral simulations, we show that the neutral chromophore (called the protonated state) and a pre-twisted derivative relax barrierlessly on a sub-500 fs timescale *via* a *Z*–*E* isomerisation pathway. This relaxation proceeds with minimal involvement of the phenyl-ring torsion coordinate that is central to the photophysics of the deprotonated, anionic chromophore. Although the internal conversion pathway passes through a twisted charge-transfer region, there is no distinct intermediate charge-transfer state in solution; instead, the dominant picosecond transients arise from cooling of a hot ground-state product. Explicit solvation calculations reveal that solvent stabilisation brings the crossing region into close proximity with the twisted coordinate, bypassing a metastable twisted charge-transfer intermediate. The combined ultrafast electronic and vibrational spectroscopy and computational modelling strategy provides a practical framework for distinguishing hot ground state cooling from excited-state intermediates when interpreting picosecond signals in fluorescent protein photoswitches and other photoisomerisable molecules.

## Abstract 2

The neutral GFP chromophore is the photoactive form in many photoconvertible and photoswitchable fluorescent proteins, yet experimental characterisation of its ultrafast dynamics requires clear assignment of the associated transient signals.

## Graphical abstract

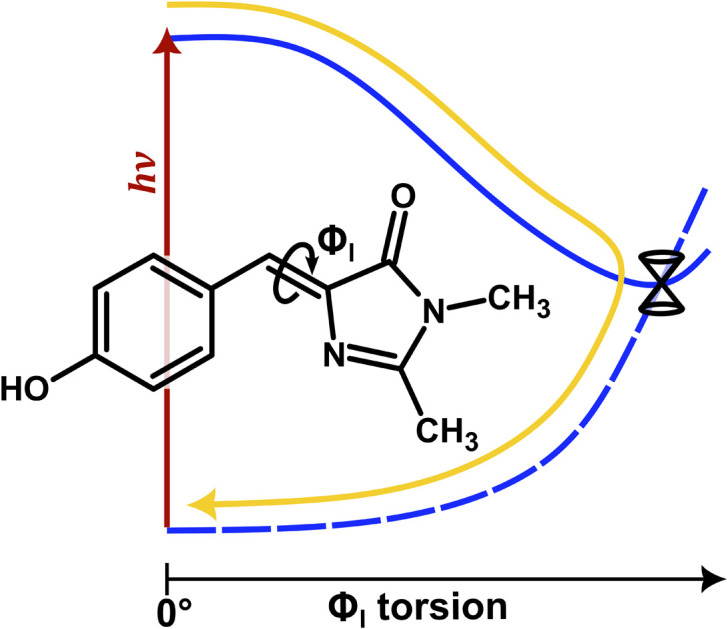

## 1 Introduction

**Fig. 1:**
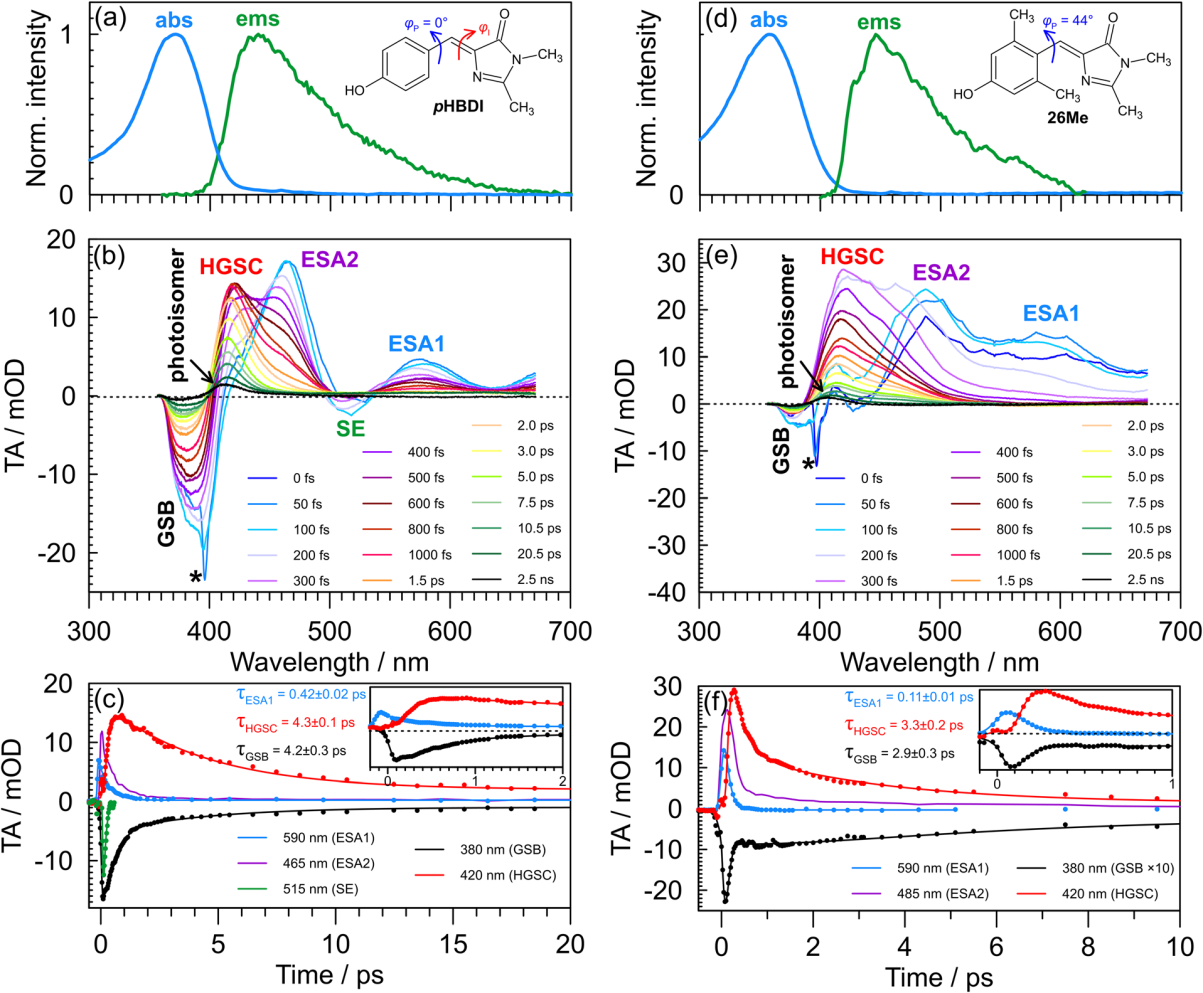
Transient absorption (TA) spectroscopy in methanol: (a) normalised absorption and fluorescence spectra (using 360 nm excitation) of *p*HBDI. (b) Selected TA spectra showing GSB, SE, ESA1, ESA2, and HGSC bands of *p*HBDI. The excited state absorptions leading to ESA1 and ESA2 were modelled and are described later. (c) Selected wavelength kinetics for *p*HBDI (points) along with kinetic fits (lines). (d) Normalised absorption and fluorescence spectra of 26Me. (e) Selected TA spectra showing GSB, ESA1, ESA2, and HGSC bands of 26Me. (f) Selected wavelength kinetics for 26Me (points) along with kinetic fits (lines) – note that GSB has been scaled by ×10. In (b) and (e), * denotes a coherent artefact. In (c) and (f), the insets show magnification over the first two picoseconds. TA spectroscopy data in acetonitrile are given in the SI. Raman scattering artefacts from solvent in fluorescence spectra have been subtracted.

Discovered in *Aequorea victoria*,^1,2^ green fluorescent protein (GFP) has become an indispensable tool in molecular and cell biology for non-invasive, real-time visualisation of cellular processes.^3,4^ The ability to genetically encode GFP has spurred the development of a broad colour palette of FPs for multicolour imaging and Förster resonance energy transfer studies, while excited-state reactivity led to the development of photoswitchable and photoconvertible FPs.^5–7^ The unique photophysics and excited-state dynamics of GFP arise from a chromophore based on the *Z* isomer of *p*-hydroxybenzylidene-imidazolinone (*p*HBDI, Fig. 1a),^8^ embedded within a rigid β-barrel structure.

In wild-type GFP, the chromophore exists in two protonation states: (i) the neutral (termed protonated) form *p*HBDI, and (ii) the anionic (deprotonated) form *p*HBDI^−^.^9,10^ In the wild-type photocycle, excitation of the neutral chromophore under physiological pH conditions initiates an excited-state proton transfer, rapidly populating the emissive anionic form,^11–13^ which has served as the primary focus of most studies. Under acidic conditions, or upon selective excitation when both forms coexist, the excited-state neutral chromophore can be prepared directly, but it fluoresces only weakly and relaxes predominantly non-radiatively.^14^ Its photophysics therefore represent a distinct excited-state manifold. GFP mutants and, notably, reversibly switchable and photoconvertible FPs, such as dronpa,^15–17^ kaede,^18^ rsEGFP,^19,20^ and Dreiklang^21,22^ rely on the neutral chromophore.

Important computational studies by Martínez and co-workers^23–25^ located conical intersections in neutral *p*HBDI with explicit water molecules and subsequently used QM/MM dynamics with microhydration to show that solvation strongly reshapes the neutral chromophore excited-state landscape. Specifically, solvent stabilises the twisted charge-transfer crossing region en route to isomerisation, thereby accelerating non-radiative decay compared with the gas-phase case. However, direct experimental tests of this picture, and clear spectroscopic assignment of the resulting transient signals, are needed.

Much of our current understanding of GFP chromophore photophysics has been developed from studies of the anionic form, *p*HBDI^−^, including gas-phase,^26–32^ solution,^33,34^ and non-adiabatic molecular dynamics (NAMD) theoretical studies.^35–38^ These works have established a conceptual framework in which excited-state dynamics are governed by two torsional reaction coordinates: phenyl-ring rotation about the bridge single bond (*φ*_P_) and imidazolinone rotation about the methylene double bond (*φ*_I_), as shown in Fig. 1a. Together with the neutral chromophore calculations discussed above, these studies suggest a common mechanistic principle: charge-transfer character, conical-intersection accessibility, and environmental stabilisation are strongly coupled in GFP chromophore photoisomerisation. However, the balance between the *φ*_I_ and *φ*_P_ pathways depends strongly on protonation state and environment. Chemical modification strategies have been used to bias these competing torsional pathways.^37,39,40^ The success of this framework has encouraged its application to derivative GFP chromophore photophysics, although the extension to neutral chromophores has not been established. Ultrafast measurements on *p*HBDI and a pre-twisted derivative (26Me) have revealed excited-state lifetimes for the neutral chromophore that are significantly shorter than those of the anion,^34,41^ suggesting that the balance between torsional motion and other excited-state dynamics differs between protonation states, requiring a new or modified framework. However, the greater multiconfigurational character of the low-lying excited states in the neutral chromophore compared with the anion has limited theoretical studies.^42–45^

More generally, a key question in ultrafast photoisomerisation is whether picosecond transient signals arise from a metastable twisted charge-transfer (TICT) intermediate,^46,47^ or from hot ground-state cooling (HGSC) following rapid internal conversion.^48–50^ In HGSC, internal conversion forms a distorted, vibrationally excited ground-state photoproduct, and the transient spectrum evolves in intensity and shape as this product cools in the solvent over a few picoseconds.^51–53^ Discriminating TICT from HGSC is therefore central to mechanistic assignment in photoswitches, and is usually only qualitatively rather than quantitatively understood, thus limiting interpretation of the measured kinetics. Gas-phase calculations have indeed suggested a TICT-like state in the photoisomerisation mechanism of *p*HBDI^−^ and anionic derivatives,^43,54,55^ although the transferability of this understanding to neutral GFP chromophores is unclear without experimental proof.

Here, we show that the neutral GFP chromophore (*p*HBDI) and a pre-twisted derivative (26Me) undergo essentially barrierless internal conversion in solution on a sub-500 fs timescale *via Z*–*E* isomerisation. Crucially, this pathway does not require the phenyl-ring torsion central to the anionic chromophore picture, and the dominant picosecond signatures arise from HGSC of a distorted photoproduct rather than from a distinct intermediate TICT state. TR-IR spectroscopy combined with anharmonic modelling allows the HGSC dynamics and spectral band shape evolution to be quantified. We show that explicit incorporation of solvent molecules is critical for modifying gas-phase potential energy surfaces and allowing experiment to be reconciled with theory.

## 2 Methods

Complete details of the experimental and theoretical methods are given in the SI. In brief, transient absorption (TA) spectroscopy was performed using the instrument described in ref. 56 with 385 nm excitation and samples loaded into a quartz flow cuvette. Time-resolved infrared (TR-IR) spectroscopy in deuterated solvents with 360 nm excitation was performed on the ULTRA LifeTime system at the Central Laser Facility, Research Complex at Harwell, Rutherford Appleton Laboratory, UK.^57^ Electronic structure calculations and NAMD trajectory simulations were performed using mixed-reference spin-flip time-dependent density functional theory (MRSF-TDDFT)^58,59^ at the BH&HLYP/6-31G* level of theory,^60,61^ implemented in GAMESS-US (July 2024 R2 release) and OpenQP 1.0.^62,63^ GAMESS-US was used for NAMD. Explicit-solvent environments were generated using the DOCKER-XTB algorithm^64^ with ORCA 6.1.0.^65^

## 3 Results and discussion

### 3.1 Ultrafast dynamics

Both *p*HBDI and 26Me in methanol (Fig. 1) and acetonitrile (SI) have strong absorption bands spanning 300–420 nm, with maximum absorption at 371 nm (*p*HBDI) and 351 nm (26Me). The *p*HBDI*Z*–*E* photoisomerisation quantum yield over the S_1_ band has been measured at 10–15% in methanol and 40–50% in acetonitrile.^66^ Transient absorption (TA) spectra of *p*HBDI (Fig. 1b) display two excited-state absorption bands (ESA1 & ESA2), a stimulated emission (SE) band, and a prominent longer-lived positive absorption on the red edge of the ground-state bleach (GSB). We assign this longer-lived band, which dominates the TA spectra after ≈ 0.5 ps, to hot ground-state cooling (HGSC); this assignment is supported independently by TR-IR measurements and spectral simulations (below). Simultaneous fitting of selected kinetic traces at wavelengths where band overlap is minimised used a sequential model, 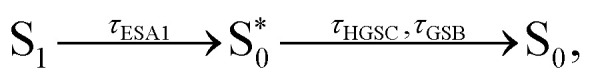 where 
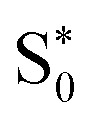
 denotes the HGSC ensemble. The GSB band kinetics required two exponential recoveries, with the first lifetime equivalent to that for ESA1 decay. The simultaneous fit excluded ESA2 band, which appears to decay with the same rate as the ESA1 band, although it shows band shape evolution presumably because of spectral overlap with SE and HGSC bands.^48^ The fit shows that ESA1 decays within ≈ 0.4 ps in both solvents, while the HGSC band decay and GSB band recovery occurs over several picoseconds (Table 1); incomplete recovery of the GSB reflects formation of a persistent photoisomer. The spectra show the persistence of incomplete GSB recovery out to ≈ 2 ns, consistent with photoisomer formation. The fitted lifetime for SE at 0.11 ± 0.02 ps is somewhat shorter than ultrafast fluorescence up-conversion measurements in ethanol,^34^ because fluorescence is weak and the SE band strongly overlaps with ESA2 and HGSC bands.

**Table 1:** Lifetimes (*τ* in picoseconds) from ultrafast transient absorption (TA) and time-resolved infrared (TR-IR) spectroscopies. *τ*_F_ are fluorescence up-conversion lifetimes in ethanol from ref. 34 with fit amplitudes given in square parentheses

Species	*τ*_ESA1_ (TA)	*τ*_ESA1_ (TR-IR)	*τ*_GSB_ (TA)	*τ*_GSB_ (TR-IR)	*τ*_HGSC_ (TA)	*τ*_HGSC_ (TR-IR)	*τ*_F_
*p*HBDI·CH_3_OH	0.42 ± 0.03^a^	0.4 ± 0.2^c^	4.2 ± 0.4	7 ± 1	4.2 ± 0.1	6 ± 1	0.18[0.54], 0.41[0.46]
*p*HBDI·CH_3_CN	0.46 ± 0.02^b^	—	5.8 ± 0.8	—	6.5 ± 0.5	—	—
26Me·CH_3_OH	0.11 ± 0.01	≈0.2^d^	2.9 ± 0.3	7 ± 1	3.3 ± 0.2	6.8 ± 0.5	0.09[0.96], 1.36[0.04]
26Me·CH_3_CN	0.15 ± 0.02	≈0.2^d^	—e	6.7 ± 0.3	3.9 ± 0.3	6.1 ± 0.8	—

a Consistent with 0.4 ± 0.2 from ref. 41.

b Consistent with 0.5 ± 0.2 from ref. 41.

c Estimated from band reshaping over the hot C=O stretch region transient (see C=O HGSC in Fig. 2a).

d Limited by cross correlation.

e GSB band is blue shifted beyond the probe spectral range.

Our data are qualitatively consistent with earlier, lower time resolution measurements.^41^ However, there are differences in band amplitudes, which we found to be due to product accumulation. Under lower flow rates, our spectra approach the earlier data and gave different kinetic fit results than the high flow rate data.

For the pre-twisted derivative 26Me, TA spectra show the same ESA1, ESA2, and HGSC bands but no resolved SE band, consistent with its shorter excited-state lifetime (Fig. 1e),^34^ while the picosecond dynamics remain dominated by HGSC (Table 1). Together with the weak viscosity dependence reported for related neutral chromophores,^34,49,67^ these observations support a rapid, volume-conserving *Z*–*E* isomerisation pathway in solution. We note that correlated *φ*_I_ and *φ*_P_ torsion, reminiscent of hula-twist motion,^68^ was previously identified from QM/MM simulations on *p*HBDI in both gas-phase and microhydrated environments.^24^

TR-IR spectra for *p*HBDI and 26Me in deuterated methanol (CD_3_OD) are shown in Fig. 2. Because of the ≈ 200 fs instrument response and coherent artefacts near *t* = 0, the earliest excited-state vibrational features are not cleanly resolved; however, TR-IR is particularly well suited to tracking HGSC dynamics through time-dependent band reshaping.^50^ Band assignments for *p*HBDI were guided by FTIR spectra and anharmonic calculations (Fig. 2b) and are consistent with isotope-labelling studies.^69^ Mode *ν*_14_ is a C=C stretch mode mostly on the phenyl ring, while the slightly higher frequency band *ν*_13_ is a localised C=C stretching band on the methylene bridge. Both of these mode are displaced upon excitation and, thus, show HGSC dynamics. Significantly, the *ν*_14_ band is flanked to higher wavenumber by a 1 + 1 combination band (*ν*_55_ + *ν*_39_), which is connected with in-plane rocking modes associated with the methylene bridge, and has roughly 50% of the intensity of the *ν*_14_ band; the combination of these two modes gives rise to the main HGSC transient shown in the inset in Fig. 2a. The C=O stretch mode (*ν*_12_) in CD_3_OD is red shifted and weakened compared with calculation due to hydrogen bonding with solvent,^49^ and is flanked with several 1 + 1 combination bands. Consistently, agreement between the deuterated acetonitrile (CD_3_CN) FTIR spectrum (non-hydrogen-bonding solvent) and calculation over the C=O stretch mode, particularly for the intensity, is closer (Fig. 2b, inset). Kinetic analysis of integrated band intensities (Fig. 2c) following a similar model to TA spectroscopy yielded *τ*_HGSC_ = 6 ± 1 ps and *τ*_GSB_ = 7 ± 1 ps (Table 1), longer than the corresponding TA-derived values, consistent with the two techniques probing different aspects of HGSC (Section 3.3). As in TA spectroscopy, GSB recovery is incomplete due to photoisomer formation.

**Fig. 2:**
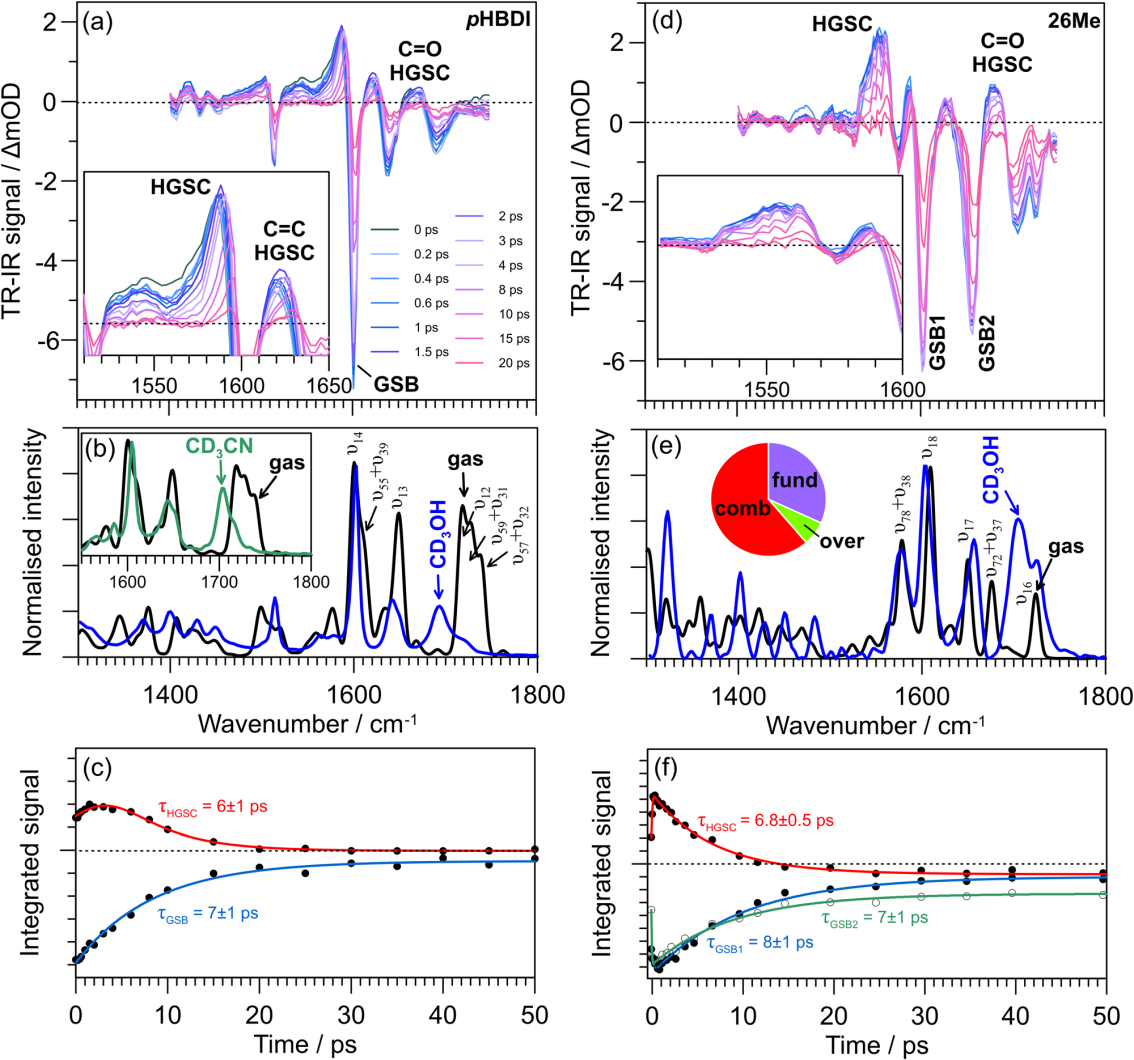
TR-IR spectroscopy in CD_3_OD: (a) selected TR-IR spectra of *p*HBDI with the C=C stretch band region shown in the inset. (b) FTIR in CD_3_OD (blue) and calculated gas phase (black) spectra for *p*HBDI, with the inset showing the FTIR spectrum in CD_3_CN (green). (c) Kinetic traces and fitted lifetimes derived from numerical integration over the strongest TR-IR bands of *p*HBDI: *ν*_14_ – phenyl ring C=C stretch, *ν*_13_ – methylene C=C stretch, *ν*_12_ – C=O stretch, *ν*_55,39_ – wag + stretch modes, *ν*_57,32,59,31,55,39_ – delocalised rock, wag, and stretch modes. (d) Selected TR-IR spectra of 26Me in CD_3_OD with the delocalised C=C stretch combination band (*ν*_78+38_) region shown in the inset. (e) FTIR in CD_3_OD (blue) and calculated gas phase (black) spectra for 26Me: *ν*_18_ – phenyl ring C=C stretch, *ν*_17_ – methylene C=C stretch, *ν*_16_ – C=O stretch, *ν*_78,38,72,37_ – delocalised stretch modes. The total IR spectrum is strongly anharmonic, with 61% of intensity arising from 1 + 1 combination bands, 32% from fundamentals, and 7% from first overtones. (f) Kinetic traces and fitted lifetimes derived from numerical integration over the strongest TR-IR bands of 26Me.

The TR-IR spectra of 26Me in CD_3_OD (Fig. 2d) show a similar HGSC band, although there are two pronounced GSB bands. Comparison with FTIR spectra and anharmonic calculations (Fig. 2e) shows that the two main bleaches are linked with C=C stretching modes (*ν*_17_ and *ν*_18_), while the main HGSC band also includes substantial contributions from the *ν*_78_ + *ν*_38_ combination band. The calculated spectrum indicates substantial combination-band intensity in this region, reinforcing the need for anharmonic treatments when interpreting the TR-IR lineshapes. Kinetic fits (Fig. 2f) following the procedure as for *p*HBDI returned lifetimes *τ*_HGSC_ = 6.8 ± 0.5 ps and ≈ 7 ps for the two GSB bands, which are consistent with *p*HBDI lifetimes and are again around twice those determined from TA spectroscopy (Table 1). The inclusion of 26Me supports the HGSC assignment for *p*HBDI in that the dynamics are not dependent on a particular vibrational marker band; despite different IR band structure and enhanced combination-band contributions, 26Me exhibits comparable picosecond reshaping and recovery kinetics consistent with general hot-product cooling rather than a metastable TICT state of *p*HBDI.

### 3.2 Potential energy surfaces & TA spectra simulation

**Fig. 3:**
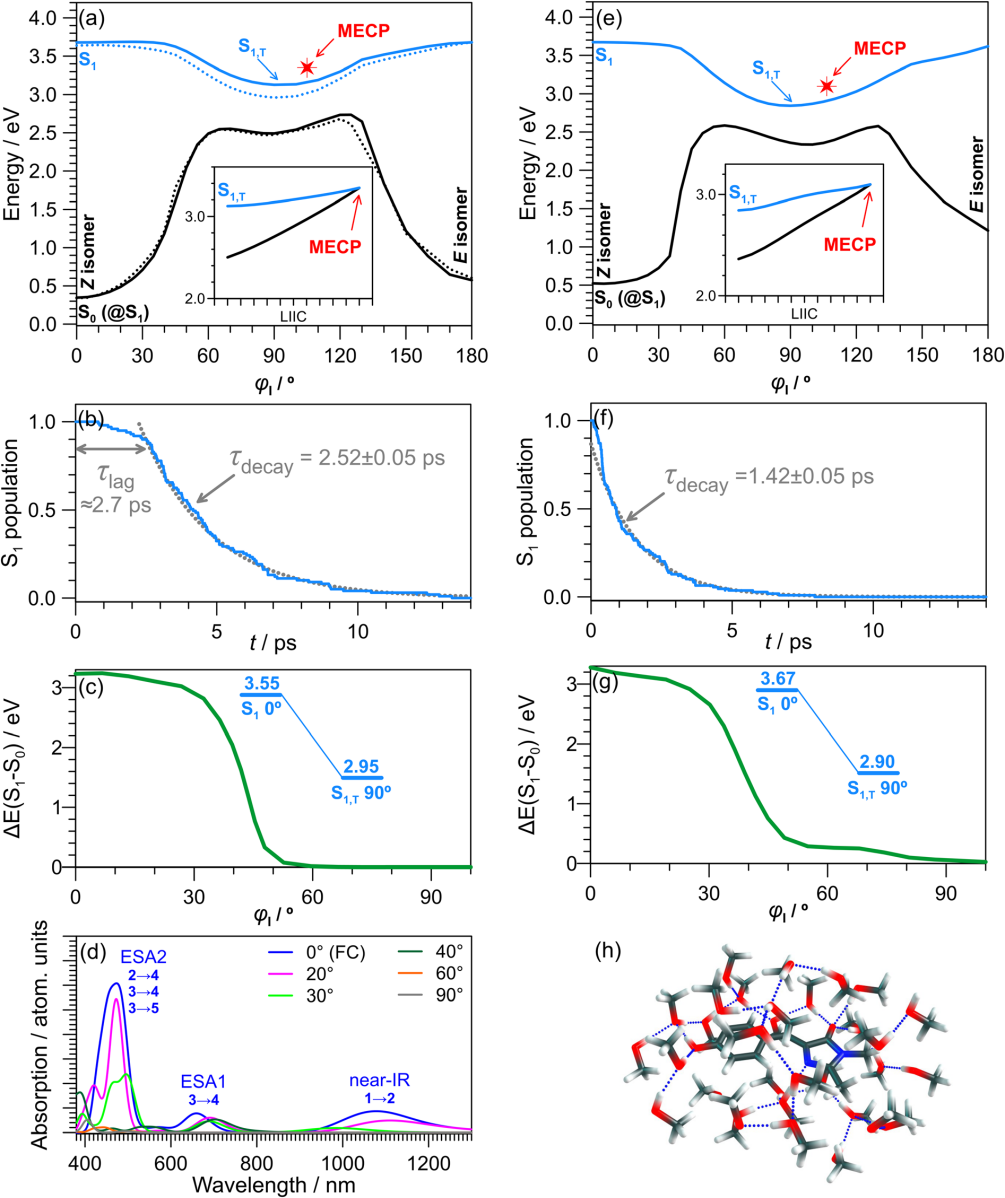
Potential energy surfaces (PESs) and gas-phase NAMD trajectories: (a) *p*HBDI relaxed PESs of the S_1_ state (solid lines are gas phase and dashed lines consider methanol implicit solvation). The inset shows the linear interpolation in internal coordinates (LIIC) between the S_1,T_ state and MECP. (b) Gas-phase NAMD trajectories for *p*HBDI show an initial lag (*τ*_lag_) due to the flat S_1_ state PES for low *φ*_I_, followed by a an exponential-like decay *τ*_decay_. The trajectories require exploration of degrees of freedom other than *φ*_I_ to reach the MECP as is situated ≈ 0.22 eV higher in energy than the S_1,T_ state geometry. (c) Explicit-methanol critical-point energies and S_0_–S_1_ energy gap along the *φ*_I_ coordinate, showing stabilisation of the twisted region and collapse of the energy gap near the MECP. Compared to (a), the solvated case is consistent with more rapid internal conversion. (d) Simulated excited state absorption spectra (see orbitals in the SI), which are consistent with experimental TA bands (Fig. 1b). (e) 26Me relaxed PESs of the (gas phase) S_1_ state. Again, the inset shows the LIIC between the S_1,T_ state and MECP with an energy difference of ≈ 0.25 eV. (f) Gas-phase NAMD trajectories for 26Me show an exponential-like decay *τ*_decay_ = 1.42 ± 0.05 ps. (g) Explicit-methanol critical-point energies and S_0_–S_1_ energy gap along the *φ*_I_ coordinate for 26Me. (h) Illustration of optimised S_1_ (*φ*_I_ = 0°) for *p*HBDI solvated with 32 methanol molecules, which is sufficient to give the first coordination shell.

The ultrafast spectroscopy indicates essentially barrierless, sub-picosecond internal conversion, which is consistent with the weak solvent friction effects reported under isoviscosity and temperature-dependent conditions and a (volume conserving) hula-twist photoisomerisation mechanism.^67^ PESs for torsion about the *φ*_I_ coordinate are shown in Fig. 3 (*φ*_P_ in the SI). In the gas phase, the S_1_ surface in both cases is essentially barrierless along *φ*_I_ to reach a twisted minimum (S_1,T_), while access to the conical intersection (MECP) requires further distortion (Fig. 3a, inset and SI). The S_1,T_ minimum is classified as a TICT state based on substantial charge separation between the two rings (SI), consistent with Martínez and co-workers.^23^ Implicit solvation calculations for *p*HBDI gave a similar topology (Fig. 3a, dashed), and *φ*_P_ torsion similarly does not produce a feasible ‘P-trap’ minimum as found for the anion.^35–37^ The gas-phase NAMD trajectories show transient trapping in the twisted region before reaching the MECP as other degrees of freedom (*e.g.* bond lengthenings) are needed to reach the MECP from the S_1,T_ state geometry, leading to a longer gas-phase lifetime (Fig. 3b) than that observed in solution. However, the absolute gas-phase lifetime from the present surface hopping trajectories should be interpreted cautiously, since earlier full multiple spawning (FMS) simulations, which include a more complete treatment of nuclear quantum effects,^24^ indicates a slightly shorter lifetime. Importantly, however, both approaches support torsional relaxation toward an *φ*_I_-twisted S_1_/S_0_ crossing.

Explicit methanol solvation (Fig. 3c, inset) stabilises the twisted region and, crucially, lowers the MECP such that it lies close in both energy and geometry to the S_1,T_ state. Consequently, trajectories in solution can undergo internal conversion more efficiently, as reflected by the S_0_–S_1_ energy gap along *φ*_I_ (Fig. 3c), where the gap collapses by *φ*_I_ ≈ 60°, accesses an extended conical intersection seam along *φ*_I_ (<1 meV). This effect can be understood from the charge-transfer character that develops upon *φ*_I_ torsion where a large difference between the S_0_ and S_1_ dipole moments develops, with the S_1_ state becoming strongly polar due to intramolecular charge transfer from the phenol ring toward the imidazolinone ring.^23,25^ Polar solvation therefore preferentially stabilises the twisted S_1_/crossing region, lowering the barrier to non-radiative decay. This solvent-induced stabilisation of the *φ*_I_-twisted charge-transfer crossing region is consistent with QM/MM trajectories,^23–25^ where FMS simulations in water predicted much faster non-radiative decay in solution (≈100 fs) than in the gas phase, with ≈ 50% of the population remained on S_1_ after ≈ 900 fs. Although direct TR-IR measurements in water are precluded by the low solubility of *p*HBDI and the limited mid-IR transparency of the solvent, the same qualitative solvent-stabilisation effect is reproduced here in methanol.

The explicitly solvated PES determined in this work was used to simulate excited-state absorption spectra (Fig. 3d), providing direct comparison with experimental TA spectra (Fig. 1b). Near the Franck–Condon region, the simulations reproduce the ESA1 and ESA2 bands and predict an additional near-IR feature whose blue edge was observed in earlier measurements.^41^ With increasing *φ*_I_ the excited-state absorption features rapidly diminish, such that by *φ*_I_ ≈ 40° only weak absorption remains. These simulations therefore support assignment of the longer-lived transient band in Fig. 1b to HGSC rather than to a metastable TICT state since the latter should not show substantial excited-state absorption over the observation window.

The PESs for 26Me (Fig. 3e) reveal an analogous mechanistic picture. Steric interactions from the methyl substituents on the phenyl ring introduce a pre-twist (*φ*_I_ = 3.1°, *φ*_P_ = 43.7°), slightly lowering the energies of S_1,T_ state and MECP relative to *p*HBDI. Gas-phase NAMD trajectories suggest faster excited-state decay than for *p*HBDI, but are still slowed because the MECP remains energetically above the S_1,T_ state and the trajectories need to explore geometric space around this minimum to reach the conical intersection seam. With explicit solvation (Fig. 3f), the MECP is again stabilised into close proximity with the S_1,T_ state, so the TICT region becomes transitory rather than a distinct intermediate state. The absence of a metastable TICT state therefore appears to be a general feature of the neutral chromophore in solution.

In summary and consistent with earlier hydrated conical intersection studies and QM/MM dynamics,^23–25^ gas-phase PESs support a metastable TICT minimum leading to picosecond-scale trapping prior to internal conversion. In contrast, explicit solvation with methanol stabilises the conical intersection region and shifting it to closely coincide in energy and geometry with the twisted S_1_ state minimum, so the TICT configuration becomes a transitory region leading to rapid, sub-picosecond internal conversion.

### 3.3 Hot ground state cooling

The lifetimes for HGSC obtained from TA and TR-IR spectroscopies systematically differ by more than the stated uncertainties (Table 1), as also reported for the related cyan fluorescent protein chromophore.^49,50^ This discrepancy is expected because the two techniques probe different observables during relaxation of a hot ensemble, and need not yield identical apparent time constants.

In electronic TA spectroscopy, for a concentration *c* of absorbing species (excited or ground state), the transient signal associated with HGSC may be expressed as

1 *I*_HGSC_ ∝ 〈*σ*(*t*)〉*c*(*t*) − 〈*σ*_300K_〉[*c*_300K_ − *c*(*t*)],

where *c*_300K_ ≫ *c*(*t*). Here, 〈*σ*(*t*)〉 is the time-dependent absorption cross-section of the cooling hot ground state population. Conventional TA analysis assumes 〈*σ*(*t*)〉 to be time invariant, so the extracted kinetics reflect only the population evolution *c*(*t*). However, during hot ground state relaxation this assumption breaks down,^48^ as large-amplitude torsional motion and substantial structural relaxation on the ground state potential energy surface lead to substantial changes in 〈*σ*(*t*)〉 with both geometry and vibrational energy content. Consequently, temperature-dependent band shifts, narrowing, and intensity redistribution during cooling can lead to apparent kinetic components unrelated to population decay.^48^ In the present case, strong spectral overlap of the HGSC band with other transient contributions (*e.g.* ESA2 and the decaying blue edge of SE at early delays) further complicates the analysis. Taking these complications together, the apparent HGSC lifetime obtained from TA spectroscopy can be biased toward the sub-population of the hot ground state that retains a large electronic absorption cross-section across the probe wavelengths, rather than reporting the full thermalisation of the ensemble.

**Fig. 4:**
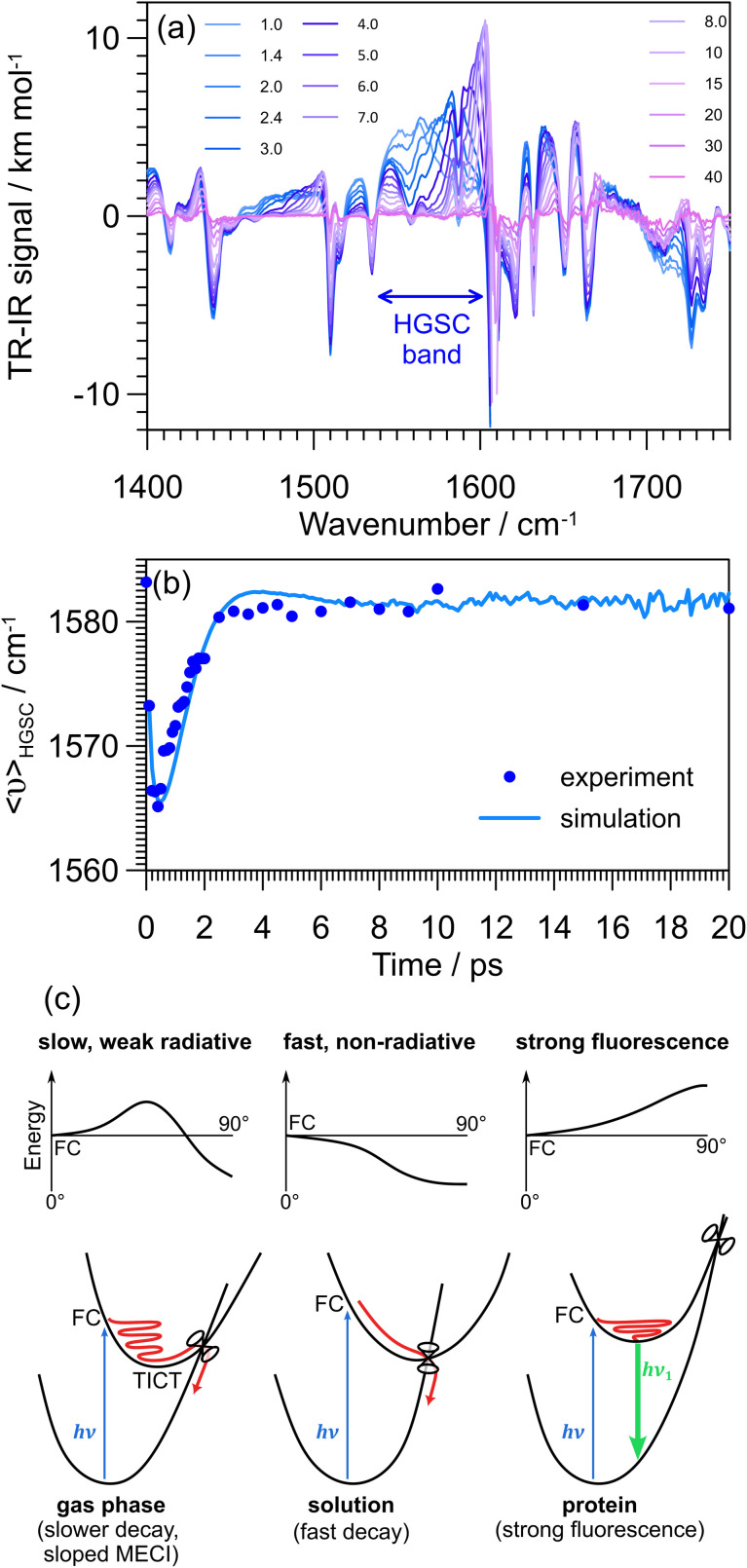
Hot ground-state cooling: (a) HGSC modelling (traces in picoseconds) of *p*HBDI using the anharmonic cascade framework.^50^ (b) Evolution of the HGSC bands compared with experiment by expectation frequency over the main HGSC band. (c) Potential outcomes for twisting about an excited-state isomerisation coordinate between Franck–Condon (FC) and a twisted state (S_1,T_). *p*HBDI and 26Me in solution align to the fast, non-radiative category. Gas phase *vs.* solution PESs highlight that solvation stabilises the twisted region leading to rapid internal conversion and no TICT state.

Ultrafast TR-IR spectroscopy is inherently more sensitive to non-equilibrium vibrational populations and anharmonic band reshaping, and therefore provides a more direct probe of HGSC dynamics (and is notably sensitive for low vibrational occupation numbers). For a hot vibrational distribution, anharmonic frequency shifts, intensity borrowing, and the appearance (or strengthening) of overtone and combination-band contributions can drive pronounced band reshaping during cooling. The TR-IR HGSC band evolution was modelled using an anharmonic cascade framework,^50^ which propagates a distribution of vibrational occupation numbers on the ground state surface and accounts for the resulting anharmonic shifts and intensity redistribution in the IR transients. The model is not intended to extract a unique initial vibrational distribution as small changes to occupation numbers will have minimal effect on the transient band evolution; rather, to see if the observed band reshaping is consistent with HGSC of modes populated by the internal-conversion geometry.^50^ For *p*HBDI (Fig. 4a) HGSC produces time-dependent reshaping rather than a simple exponential amplitude decay, with the model reproducing the experimental band shape evolution (Fig. 4b) over the picosecond timescale, confirming that the dominant transient features in the ultrafast spectroscopy arise from HGSC rather than a metastable TICT state on the S_1_ state PES. This analysis illustrates why the combination of TA and TR-IR spectroscopies is useful for photoisomerising systems more generally: TA captures the coupled evolution of population and electronic absorption cross-section, whereas TR-IR provides a structurally specific probe of anharmonic vibrational cooling through band reshaping.

The relationship between gas-phase and explicitly solvated dynamics for *p*HBDI and 26Me is summarised in Fig. 4c within the methylene twisting framework of Olsen and co-workers.^70^ In the gas phase, torsion along the *Z*–*E* isomerisation coordinate accesses a TICT-like state, S_1,T_, that is separated from the conical intersection seam, leading to delayed surface crossing. In solution, explicit solvation stabilises the conical intersection region and brings it into close energetic and geometric proximity with the twisted S_1_ state region, such that internal conversion becomes effectively barrierless on the sub-picosecond timescale and there is no distinct TICT state. Because *p*HBDI exhibits a barrierless pathway along the isomerisation coordinate and the twisted geometry is non-radiative, it aligns with the fast, non-radiative scenario in Fig. 4c, consistent with the observed ultrafast loss of excited-state transients in TA spectroscopy (and fluorescence upconversion). In non-photoconvertible fluorescent proteins such as wild-type GFP, electrostatic, hydrogen bonding, and steric interactions between amino acid residues and the *p*HBDI chromophore restricts double-bond torsion, inhibiting internal conversion and allowing fluorescence.^2,33^ There, the dynamics follow the right-hand case in Fig. 4c where the twisted region is energetically unfavourable.

## 4 Conclusions

**Fig. 5:**
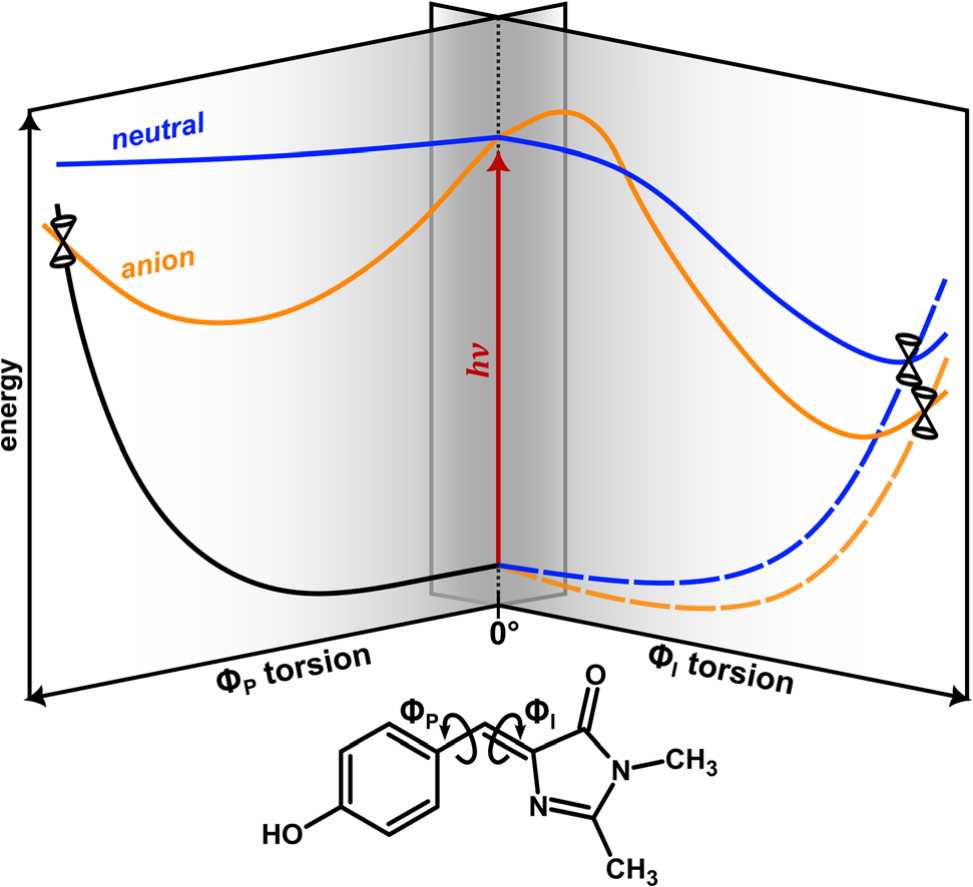
Summary of neutral (*p*HBDI, blue) and anion (*p*HBDI^−^, orange) model GFP chromophore potential energy surfaces in solution. The neutral has a single internal conversion pathway, while the anion involves a single-bond twist and ‘P-trap’ (*φ*_P_ coordinate) due to weakening of the bridge single bond.^35,36^ In the neutral, relaxation proceeds along the methylene torsional coordinate (*φ*_I_), while in the anion the P-trap can temporarily delay internal conversion, leading to extended excited-state lifetimes. The GFP chromophore protonation state therefore adjusts the balance between methylene double-bond isomerisation and phenyl-ring twisting.

The protonated state of the GFP chromophore in solution undergoes a barrierless ultrafast relaxation on a sub-500 fs timescale *via Z*–*E* isomerisation about the methylene bridge, with minimal involvement of the phenyl-ring torsion central to the dynamics in the anionic chromophore rendering single-bond torsion a non-competitive pathway. The key implication is interpretive rather than merely kinetic: the dominant picosecond transient features arise from formation and cooling of a vibrationally hot ground-state photoproduct, not from a distinct TICT intermediate state. Thus, the neutral chromophore follows a simplified excited-state pathway with rapid access to a single crossing region. These PESs compared with the anionic case are summarised in Fig. 5.

Explicit solvation calculations revealed that solvent interactions are not a passive perturbation but actively reshape the non-radiative pathway by stabilising the double-bond twisted region and bringing it into close energetic proximity to the conical intersection seam, enabling rapid internal conversion and making the twisted region transitory in solution. In contrast, the gas phase case (and implicit solvation models) show temporary trapping in the twisted region, highlighting the need for explicit solvation when connecting (gas phase) computed topologies to condensed-phased spectroscopic measurements. This behaviour follows the general principle found by Martínez and co-workers^23,25^ that polar environments can tune access to charge-transfer-mediated conical intersections: as *φ*_I_ torsion increases, the charge-transfer character of the S_1_ state potential energy surface, solvent electrostatics preferentially stabilise the crossing region and convert a potentially metastable TICT minimum into an efficient doorway for internal conversion.

More broadly, our results emphasise that TA kinetics do not necessarily report pure population dynamics during hot ground-state cooling. Evolving band shapes and absorption cross-sections, especially under spectral overlap, can produce apparent time constants that differ from those obtained by ultrafast vibrational spectroscopy. Here, the ultrafast vibrational spectroscopy provides the clearest fingerprint of hot ground state cooling through time-dependent band reshaping, whereas TA spectroscopy tends to reflect the combined evolution of electronic absorption cross-sections and population dynamics. Combining TA and TR-IR spectroscopies therefore offers a practical strategy for disentangling sub-picosecond electronic relaxation from ensuing picosecond thermalisation in photoswitches and photoisomerising molecules.

## Author contributions

TA spectroscopy was performed by AF under the supervision of SRM and JNB, and TR-IR spectroscopy was performed by AF at the Rutherford Appleton Laboratory ULTRA facility supported by PM. Spectrophotometry and FT-IR was performed by EKA, JAW, and JNB. QT sampling for the NAMD trajectories was performed by PC and JNB. MRSF-TDDFT calculations were performed by JNB and EKA and were discussed with WP and CHC. HGSC modelling of TR-IR spectra and associated method development was performed by MHS and JNB. Experimental and theoretical data were interpreted by AF and JNB, and were discussed with all authors. The project was managed by JNB. The manuscript was prepared by JNB and was discussed by all authors.

## Conflicts of interest

There are no conflicts to declare.

## Supplementary Material

SC-017-D6SC02114J-s001

## Data Availability

The data that support the findings of this study are available from the corresponding author upon reasonable request. Supplementary information (SI): experimental and computational methods; TA spectroscopy of *p*HBDI and 26Me in acetonitrile; TR-IR spectroscopy of 26Me in CD_3_CN; PES for *φ*_P_ torsion; Löwdin charges with *φ*_I_; analysis of NAMD trajectories; orbitals involved in S_*n*_ ← S_1_ absorption calculations; HGSC modelling of 26Me; selected critical point geometries; animated gif images of selected vibrational modes. See DOI: https://doi.org/10.1039/d6sc02114j.
