# Supplementary material for: GFP chromophore photophysics: ultrafast dynamics and hot ground state cooling in the neutral form
Source: Chem Sci. 2026 Jun 22;17(31):15098–107. doi: 10.1039/d6sc02114j (PMC13312905; doi:10.1039/d6sc02114j)
Supplement: Supplementary file 1 [file SC-017-D6SC02114J-s001.pdf]

Supplementary Information (SI) for Chemical Science.
This journal is © The Royal Society of Chemistry 2026

Electronic Supplementary Information for

GFP Chromophore Photophysics: Ultrafast Dynamics and Hot Ground State Cooling in the Neutral Form

Anam Fatima,[a] Mark H. Stockett,[b] Eleanor K. Ashworth,[a] Woojin Park,[c] Cheol Ho Choi,[c] Joseph A. Wright,[a] Pratip Chakraborty,[a] Partha Malakar,[d] Stephen R. Meech,[a] and James N. Bull[a]

[a] Chemistry, Faculty of Science, University of East Anglia, Norwich NR4 7TJ, United Kingdom
[b] Department of Physics, Stockholm University, SE-10691 Stockholm, Sweden
[c] Department of Chemistry, Kyungpook National University, Daegu 41566, South Korea
[d] Central Laser Facility, Research Complex at Harwell, Rutherford Appleton Laboratory, Didcot OX11 0QX, United Kingdom

Contents

1 Methods **S3**

1.1 Experimental . . . S3

1.1.1 Steady-state spectroscopy . . . S3

1.1.2 TA spectroscopy . . . S3

1.1.3 TR-IR spectroscopy . . . S3

1.2 Computational . . . S3

1.2.1 Potential energy surfaces . . . S3

1.2.2 Vibrational frequencies . . . S4

1.2.3 QT sampling & NAMD trajectories . . . S4

1.2.4 HGSC modelling . . . S4

2 TA spectroscopy in acetonitrile **S6**

3 TR-IR spectroscopy of 26Me in CD_3CN **S7**

4 PES for φ_P torsion **S8**

5 Löwdin charges and TICT state **S9**

6 Analysis of NAMD trajectories **S10**

7 Orbitals involved in S_1 absorption calculations **S11**

8 Solvated potential energy surfaces **S12**

9 Optimised geometries of critical points **S13**

1 Methods

1.1 Experimental

1.1.1 Steady-state spectroscopy

Steady-state absorption and emission spectra of ***p*HBDI** and **26Me** in methanol and acetonitrile (Merck, >99.5%) were measured using a Perkin-Elmer Lambda XLS spectrometer and an Edinburgh Instruments FS5 spectrofluorometer. FTIR spectra in deuterated solvents (Fisher 99.8% CD_3OD and CD_3CN) were recorded using a Bruker Vertex 80 spectrometer equipped with a deuterated triglycine sulfate (DTGS) detector set at a resolution of $4\,cm^{-1}$. Measurements used a liquid cell equipped with CaF_2 windows and a 1 mm optical pathlength.

1.1.2 TA spectroscopy

Ultrafast transient absorption (TA) spectroscopy at $T = 291$ K was performed on ***p*HBDI** and **26Me** (1–2 mM in methanol and acetonitrile) using the instrument detailed in Ref. 1. The pump and probe beams were derived from the 800 nm fundamental output beam from a Spectra Physics-Mai Tai laser oscillator coupled with a Ti:Sapphire regenerative amplifier (Spectra Physics Spitfire ACE). The amplified output pulses at 800 nm (≈100 fs, 1 kHz, ≈5 mJ pulse^{-1}) were directed into an optical parametric amplifier (OPA, Light Conversion TOPAS Prime), which produced 385 nm (attenuated to ≈200 nJ pulse^{-1}) pump pulse for sample excitation. A white light continuum (WLC) probe spanning 300–700 nm was generated by focussing part of the 800 nm fundamental beam onto a ≈3 mm thickness CaF_2 window that was continuously *x*–*y* translated to prevent damage to the material surface. All measurements were conducted using 1 mm pathlength quartz flow cuvettes, with the sample concentration adjusted to achieve an optical density of ≈0.5 at the excitation wavelength. A flow cuvette was necessary to avoid photoproduct accumulation; static and low flow rate sample measurements resulted in different band shapes and relative band intensities to the high flow rate measurements.

It is worth noting that global fitting of the entire TA spectra set using a sequential model in GloTarAn[2] led to an unphysical interpretation. In particular, four kinetic components were required to extract evolution-associated decay spectra (basis functions) that provided a sufficient fit to the spectra, reflecting a ‘compromise fit’ rather than distinct molecular states. This complication arises because the underlying excited-state absorptions exhibit spectral evolution with time (as expected for an ultrafast isomerising system), meaning that the TA spectra cannot be adequately represented by a linear combination of static basis spectra simply changing amplitudes with time.

1.1.3 TR-IR spectroscopy

Ultrafast TR-IR spectroscopy at $T = 293$ K on ***p*HBDI** and **26Me** (1–2 mM in deuterated methanol, CD_3OD) was performed using the ULTRA LifeTime system at the Central Laser Facility, Research Complex at Harwell, Rutherford Appleton Laboratory, UK.[3,4] The sample, loaded in a 50 μm path length CaF_2 cell, was excited with 360 nm light pulses (500 nJ pulse^{-1}, 150 μm spot size) at a 1 kHz repetition rate, and probed with a delayed IR pulse to capture transient vibrational spectra (cross correlation ≈200 fs). The sample position was rastered in two dimensions and flowed during measurements to minimise localised photobleaching and degradation. Measurements were performed with and without the pump pulse (pump-on/pump-off) under magic angle polarisation geometry. Spectral calibration used a standard polystyrene IR spectrum.

1.2 Computational

1.2.1 Potential energy surfaces

Excited state potential energy surfaces (PESs) were determined using mixed-reference spin-flip time-dependent density functional theory (MRSF-TDDFT)[5,6] as implemented in GAMESS-US (July 2024 R2 release) and OpenQP 1.0.[7,8] Importantly, MRSF-TDDFT can adequately describe PESs involving charge-transfer character and PESs near conical intersection seams;[5,9] conventional TD-DFT fails in these aspects.[10,11] Our calculations focussed on the *Z* isomer since our samples are isomerically pure, and the *Z* isomer is present in wild-type GFP. Earlier calibration calculations on (gas phase) ***p*HBDI** along the *Z*-*E* photoisomerisation coordinate found the topology for the S_0 and S_1 state with MRSF-TDDFT (BH&HLYP/6-31G*)[12,13] to be in good accord with that determined from calculations using XMS-CASPT2(10,9)/6-31G* with three-root averaging.[14] Such a large active space makes multireference calculations computationally demanding, thereby restricting the practical application of NAMD trajectory methods. The gas-phase (and implicit solvated) S_1 state potential energy surfaces along the φ_I co-ordinate were relaxed scans where φ_I was fixed and all other degrees of freedom were optimised.

Solvation effects on the potential energy surfaces were considered using an implicit SMD model for methanol,[15] or using explicit solvent molecules. For the latter case, 32 methanol molecules (as a balance between cost and accuracy) were placed with the DOCKER algorithm[16] using XTB trajectories at $T = 300$ K and the ORCA 6.1.0 package.[17–19] In turn, the solvent molecules were reoptimised (the most computationally demanding part of the study) on the S_1 state with a frozen gas-phase chromophore geometry. Next, the solvent molecules were fixed and the chromophore in the cluster was optimised. The solute-solvent cluster geometries were used to simulate the expected TA spectra using MRSF-TDDFT. The solvated $S_{1,T}$ and minimum energy crossing point (MECP) conical intersection geometries

were optimised with 15 explicit solvent molecules (32 was too computationally demanding). In turn, the remaining solvent molecules were added and the full solvation shell was reoptimised with the chromophore geometry frozen. In both cases the MECP S_1-S_0 energy gap was <1 meV. Because of the many low-frequency solvent degrees of freedom and the large number of near-degenerate local solvent configurations, the explicit-solvent data in Fig. 3c,g in the paper are presented as critical-point energies and S_0-S_1 energy gaps rather than as separate S_0 and S_1 relaxed profiles.

1.2.2 Vibrational frequencies

Anharmonic vibrational frequencies on the ground electronic state of ***p*HBDI** and **26Me** were computed with second-order vibrational perturbation theory (VPT2)[20] at the B3LYP/N07D level of theory[12,21] with Gaussian 16.B01.[22] These computations were performed using very tight optimisation criteria (1×10^{-12}) and a custom integration grid consisting of 200 radial shells and 974 angular points per shell. The N07D basis set takes 6-31G and adds selected dispersion and polarisation functions that improve the accuracy of calculated vibrational frequencies for large aromatic and highly conjugated molecules.[23] The B3LYP/N07D level of theory in the VPT2 framework has been found to give best agreement with ground-state IR spectra for conjugated and aromatic molecules compared with gas-phase IR spectra.[24–26]

1.2.3 QT sampling & NAMD trajectories

Initial geometries and velocities for the (gas phase) NAMD trajectories were obtained from ground state sampling at the BH&HLYP/6-31G* level of theory. This involved starting four sets of ground-state molecular dynamics trajectories for each species that were propagated ($\approx$0.48 fs steps) and thermalised (out to $\approx$20 ps) with a quantum thermostat (QT)[27,28] using the ABIN code[29] interfaced to ORCA 6.1.0,[19] initiated from the optimised geometry with velocities obtained from a Boltzmann distribution. The Generalised Langevin Equation (GLE) thermostat parameters (drift and diffusion matrices A and C) were collected from the GLE4MD webpage[30] for $T=298.15$ K, along with $N_s = 6$ (additional degrees of freedom), $\hbar\omega_{max}/k_B T = 20$, in the strong coupling regime. ω_{max} represents the maximum fundamental mode frequency for which the GLE parameters were optimised, for a particular temperature, and at $T=298.15$ K, it is 4114.5 cm^{-1}. This value is larger than the highest frequency fundamental mode. Equilibration time of the trajectories was determined by monitoring the convergence of the average kinetic energy temperature.[31] The initial geometries and velocities for NAMD trajectories were obtained by taking snapshots at $\approx$500 fs intervals from the thermalised portion of the QT trajectories.

NAMD trajectories were performed using MRSF-TDDFT[6] at the BH&HLYP/6-31G* level of theory in GAMESS-US. The NAMD trajectories used Tully's fewest-switches surface-hopping algorithm,[32] with non-adiabatic coupling vectors computed numerically using a fast overlap method.[33,34] Velocity Verlet was used for integration. The trajectories were propagated for 15 ps using a time step of 0.5 fs. Energy conservation during the hops was ensured by rescaling of the velocities. The nuclear degrees of freedom were propagated with a subtime-step size of 10^{-5} fs for the electronic degrees of freedom.[35,36] No corrections for decoherence were applied. 100 trajectories were initiated on the S_1 state for ***p*HBDI** (109 for **26Me**), with 1 trajectory removed for ***p*HBDI** and 3 trajectories removed for **26Me** due to SCF convergence issues.

1.2.4 HGSC modelling

HGSC transients in the TR-IR spectra for ***p*HBDI** were modelled using a recent anharmonic cascade framework.[4] The framework utilised vibrational state occupation numbers (n_i) determined from projections of the NAMD trajectories at the surface-hopping geometries and vibrational energies of two electronic states with statistical modelling of vibrational absorption and energy dissipation for each mode. Vibrational energy dissipation from the molecule to the environment was described by a single adjustable probability parameter, q, which affects only the rate of transient evolution. For ***p*HBDI**, we used $q = 0.01$.

In our earlier work,[4] the infrared intensities of the vibrational transitions were given as:

$$I_n = (n+1) * I_0 * (h\nu_n/h\nu_0),$$

with similar expressions for overtone and combination bands. For ***p*HBDI**, we found this scaling overestimated the transient absorption intensity, with the following expression giving better agreement with experiment:

$$I_n = I_0 * (h\nu_n/h\nu_0),$$

again with similar expressions for the overtone and combination bands (i.e. no scaling with occupation number). This suggests that for ***p*HBDI**, the lifetime of the vibrationally excited levels is limited by intramolecular vibrational energy redistribution (IVR) rather than the intrinsic transition dipole moment. To explicitly account for IVR, we have extended the model include IVR by proposing transfer steps in each timestep of the statistical simulation, where one quantum of vibrational energy may be exchanged between modes i and j with a probability given by a Fermi Golden Rule relationship:[37]

$$p_{trans,ij} = 4\pi c n_i dt |X_{ij}|^2 \frac{\gamma}{\Delta\nu_{ij}^2 + \gamma^2}, \quad (1)$$

where γ is the IVR-limited linewidth, $\Delta\nu_{ij} = |\nu_i - \nu_j|$, and X_{ij} are elements of the anharmonic X-matrix. We took $\gamma \approx 32$ cm^{-1} (sensible from our FTIR spectra), but this could, in principle, be constrained by higher resolution IR measurements.

2 TA spectroscopy in acetonitrile

TA spectra in acetonitrile are shown in Fig. S1. As for TA spectroscopy in methanol, selected wavelength kinetic traces (Fig. S1c) were fit in a simultaneous model: $S_1 \xrightarrow{\tau_{ESA1}} S_0^* \xrightarrow{\tau_{HGSC}\,/\,\tau_{GSB}} S_0$, where S_0^* is the hot ground state. The TA spectra in acetonitrile show significant enhancement of the ESA1 band compared with in methanol.

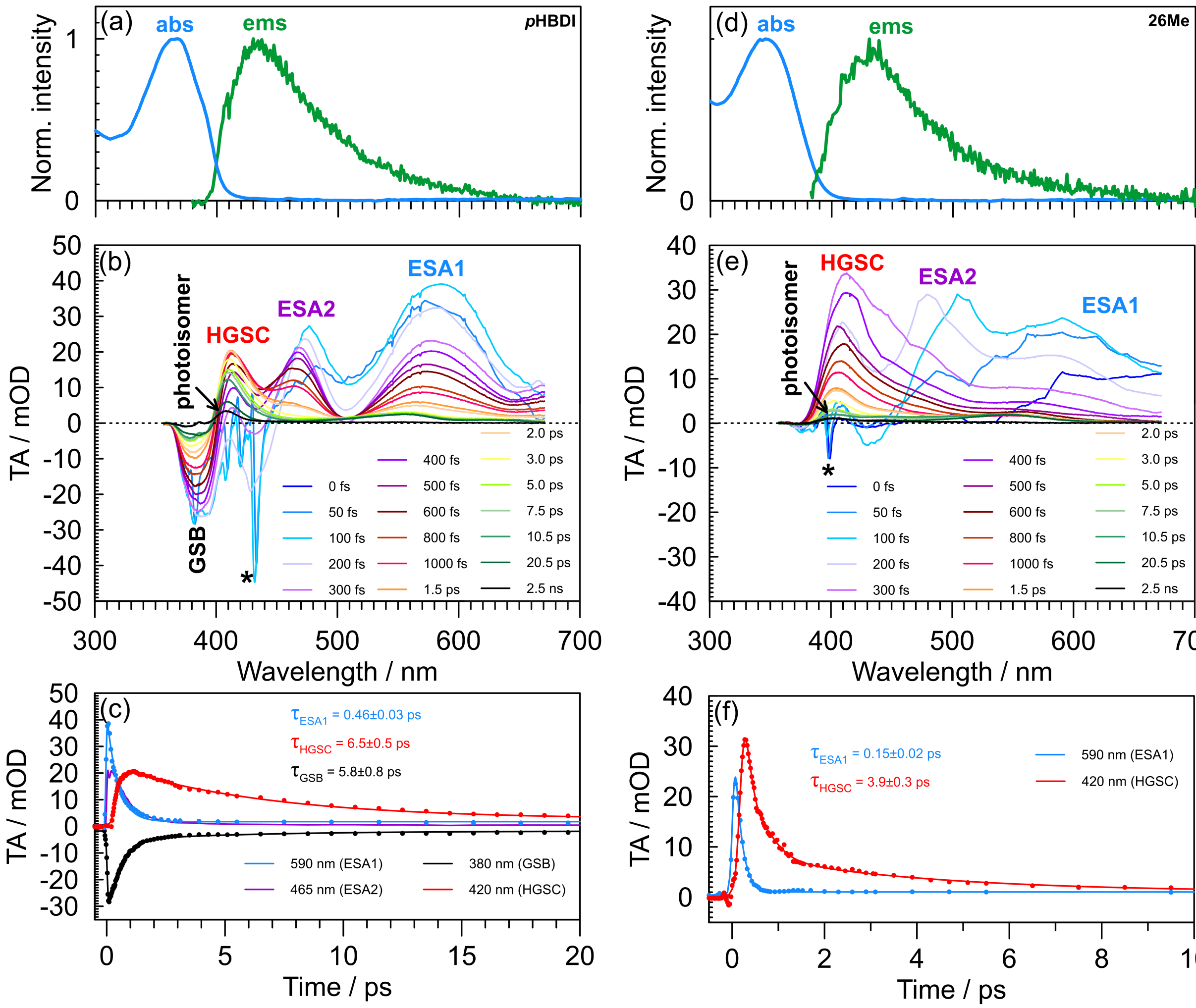

Fig. S1 Transient absorption (TA) spectroscopy in acetonitrile: (a) Normalised absorption and fluorescence spectra (using 360 nm excitation) of pHBDI. (b) Selected TA spectra showing GSB, SE, ESA1, ESA2, and HGSC bands of pHBDI. (c) Selected wavelength kinetics for pHBDI (points) along with kinetic fits (lines). (d) Normalised absorption and fluorescence spectra of 26Me. (e) Selected TA spectra showing GSB, ESA1, ESA2, and HGSC bands of 26Me. (f) Selected wavelength kinetics for 26Me (points) along with kinetic fits (lines). In (b) and (e), * denotes a coherent artefact.

3 TR-IR spectroscopy of 26Me in CD_3CN

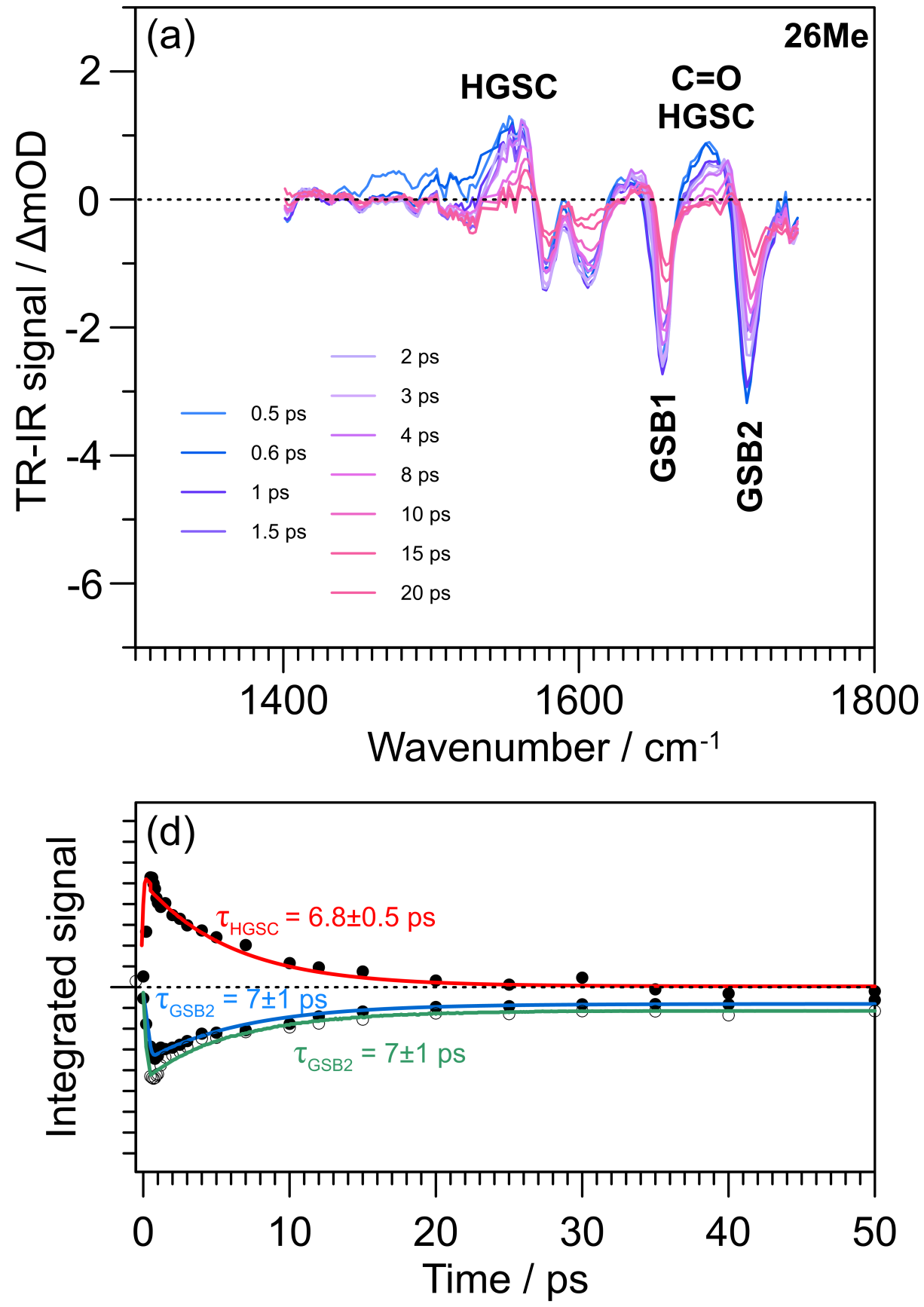

Fig. S2 TR-IR spectroscopy of 26Me in CD_3CN: (a) Selected TR-IR spectra. (b) Kinetic traces and fitted lifetimes derived from numerical integration over the strongest TR-IR bands.

4 PES for φ_P torsion

Relaxed PES of the S_1 state with rotation about φ_P are shown in Fig. S3. For both ***p*HBDI** and **26Me** there is no P-trap (distinct potential energy minimum) with φ_P that could temporarily trap excited-state population. The lack of this coordinate, which is central to the excited-state dynamics in ***p*HBDI**$^-$, leads to reduced excited-state lifetimes for the neutral form. Note that **26Me** has a pre-twist on the S_0 state due to steric interactions (discussed in the paper).

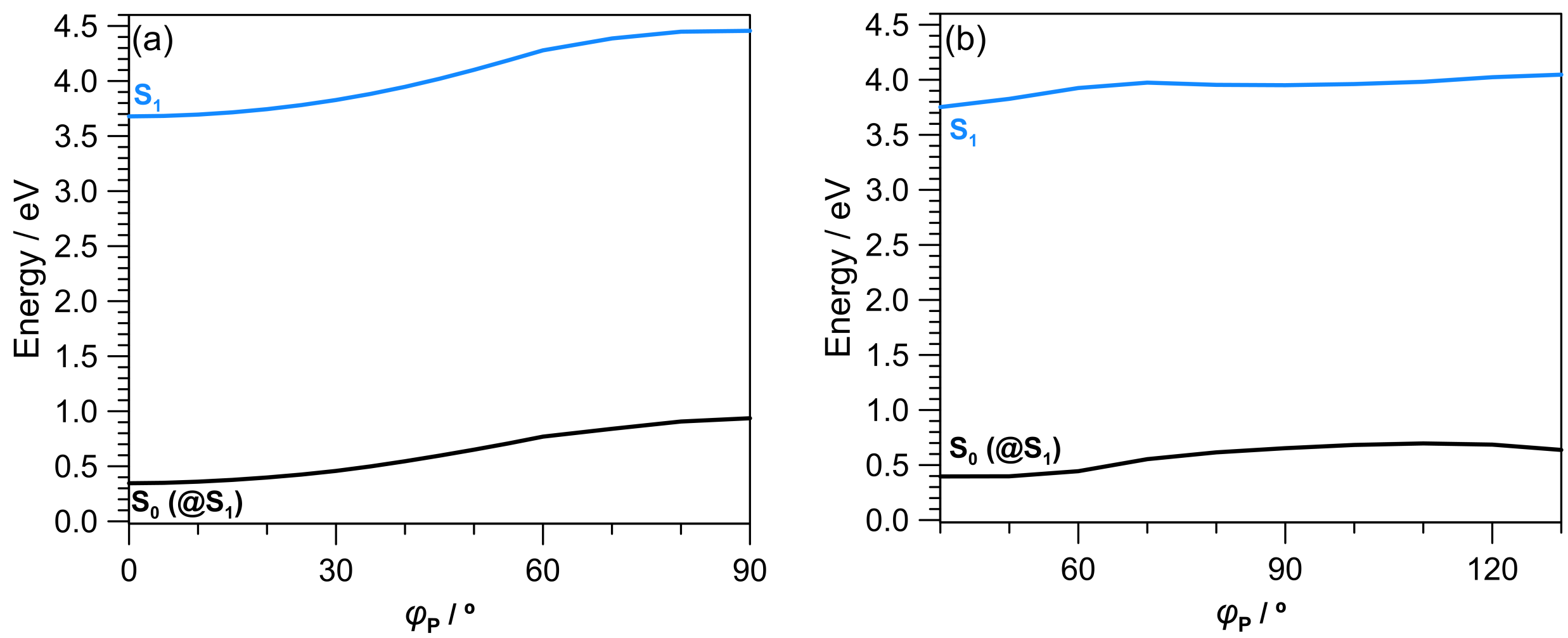

Fig. S3 Relaxed potential energy surfaces (PESs) for torsion about the φ_P coordinate: (a) pHBDI PESs with geometric optimisation of the S_1 state. (b) 26Me PESs assuming geometric optimisation of the S_1 state.

5 Löwdin charges and TICT state

The charge-transfer character of the gas-phase PES (Fig. S4) with φ_I torsion, was quantified through Löwdin charges on each ring system.[38] Because the $S_{1,T}$ state has $\varphi_I \approx 90°$ and there is a substantial difference in charges between the two ring systems, $S_{1,T}$ is classified as a TICT state in the gas phase.

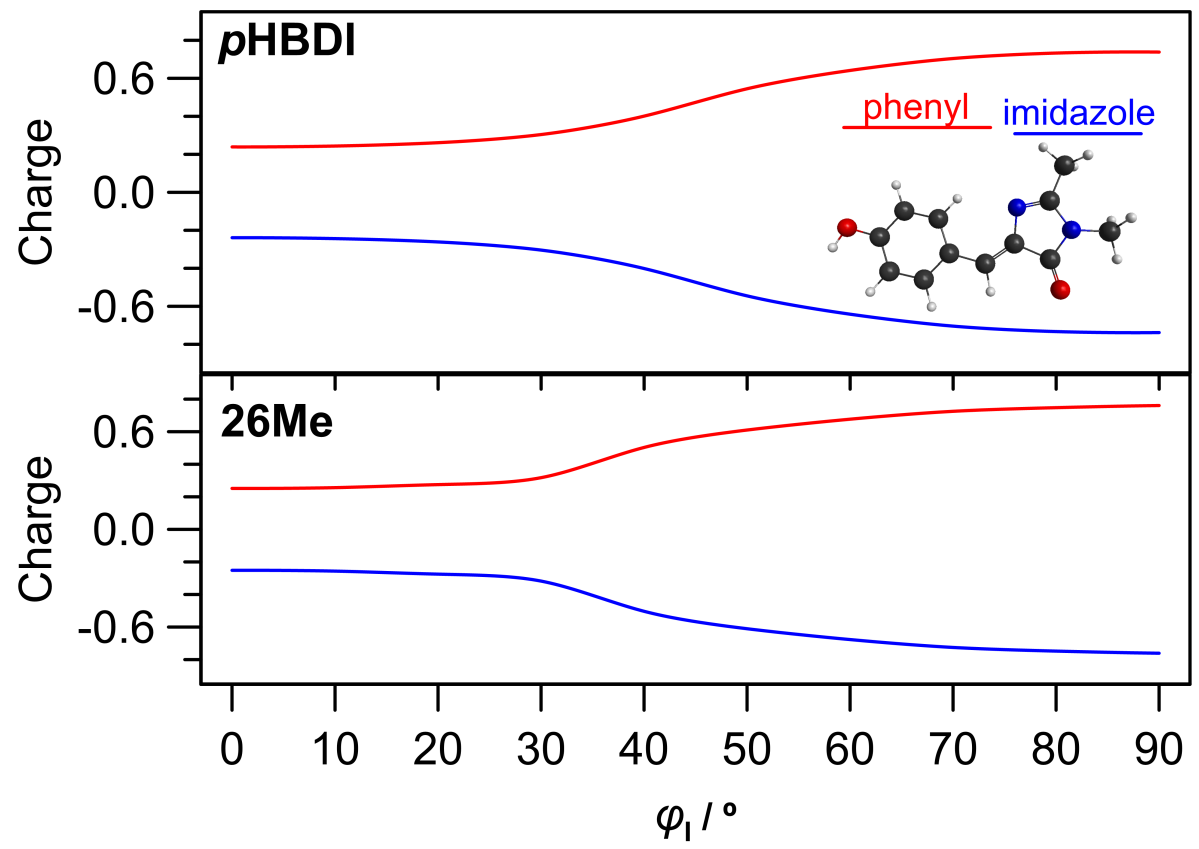

Fig. S4 Löwdin charges on each ring system along the relaxed S_1 potential energy surfaces with φ_I torsion.

6 Analysis of NAMD trajectories

Geometries of the critical points for the gas-phase PES are shown in Fig. S5 along with a summary of the surface-hopping coordinates for each trajectory. The three dihedral angles are defined as: φ_I atoms 1-2-3-5, φ_{HOOP} (hydrogen out-of-plane) atoms 1-2-3-4, and φ_P atoms 6-5-3-2. Surface hopping occurs for $\varphi_I \approx 80-110°$, while the HOOP mode and φ_P play little role (surface hoppings are distributed about initial equilibrium values).

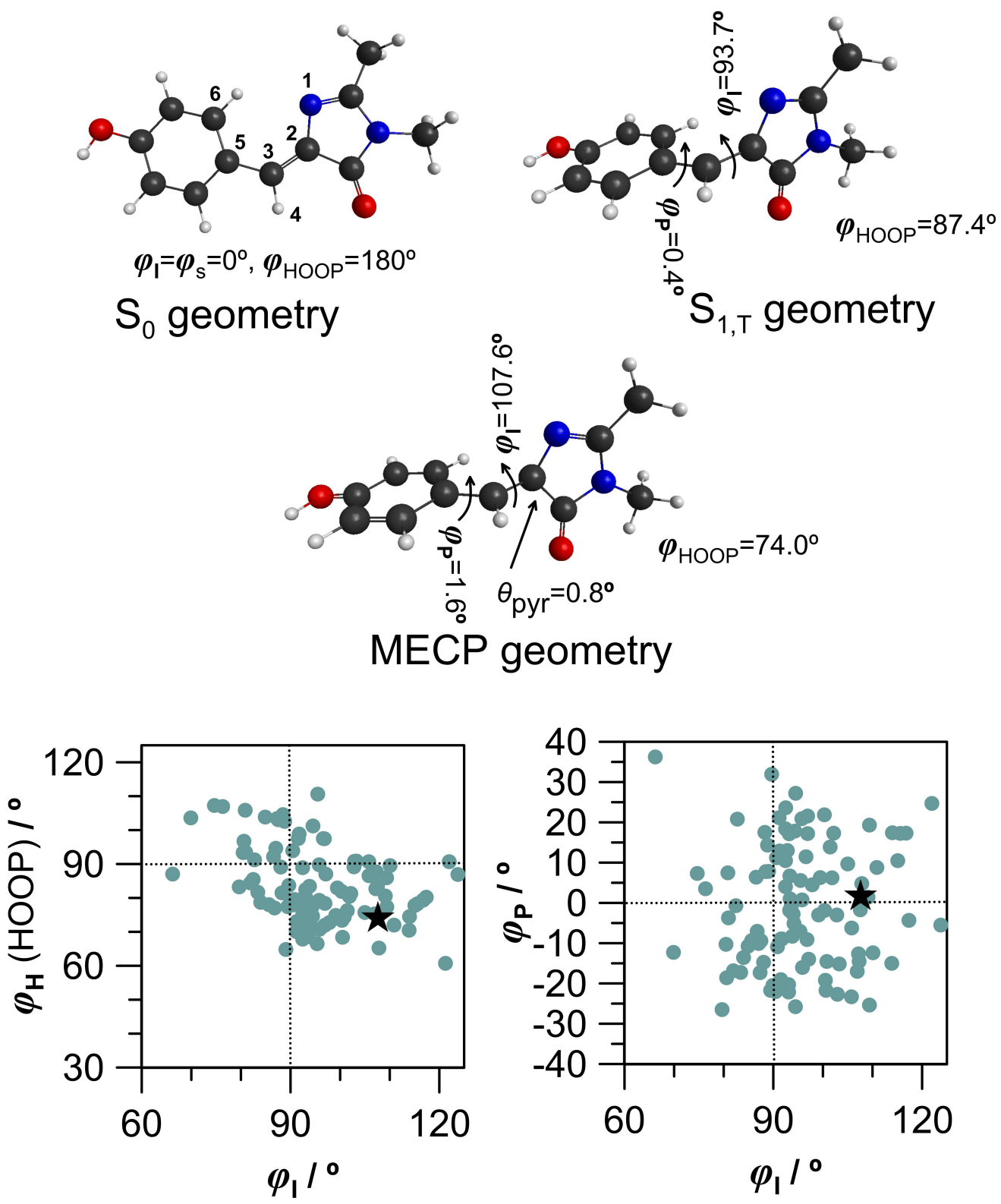

Fig. S5 Critical point illustrations and NAMD trajectories for pHBDI. The lower two plots show surface hopping geometries for the trajectories, with the MECP given as the black star.

7 Orbitals involved in S_1 absorption calculations

The orbitals shown below are those associated with the transitions in the modeled excited-state absorption spectra for solvated ***p*HBDI**.

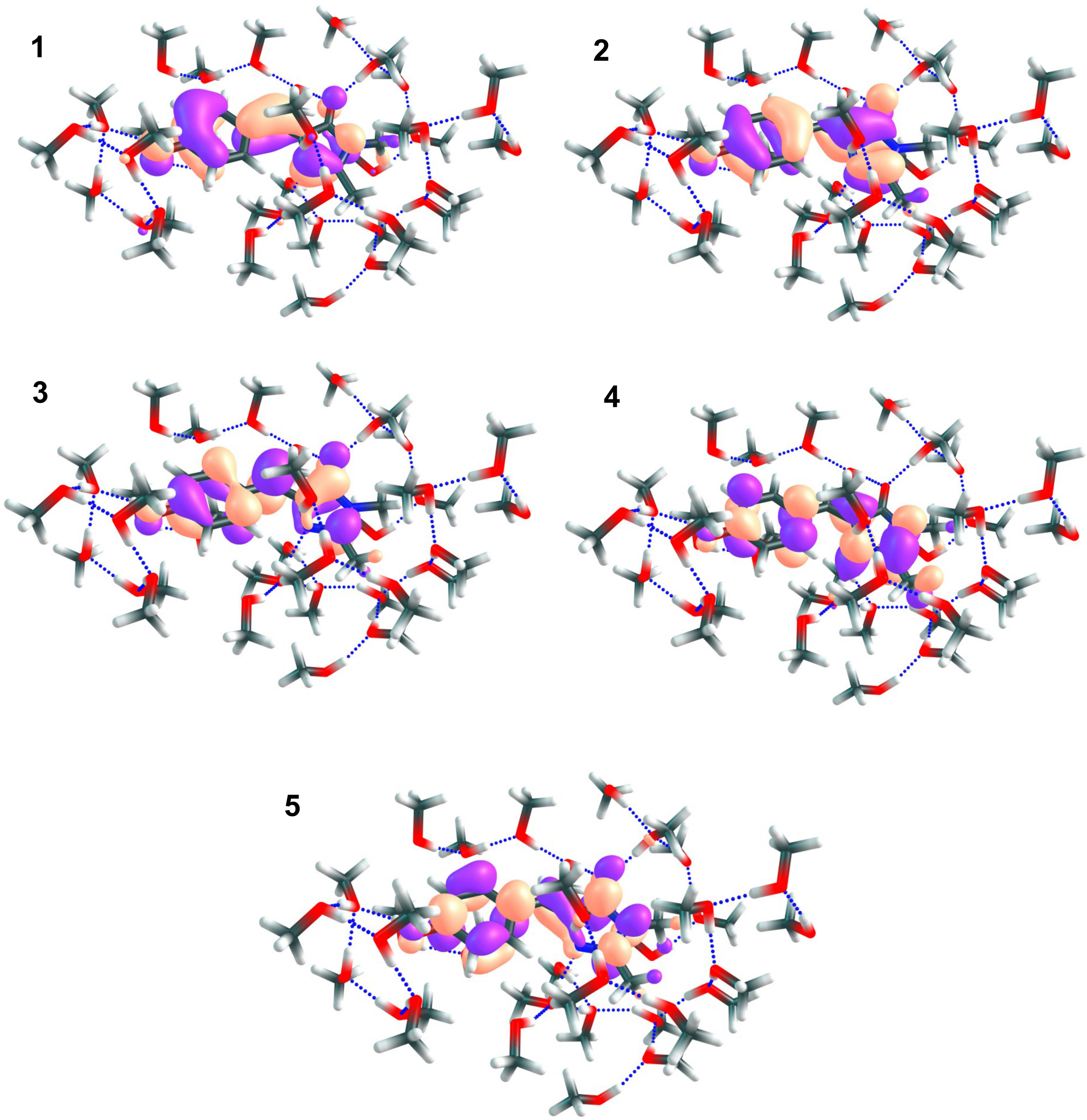

8 Solvated potential energy surfaces

The relaxed potential energy surfaces calculated with explicit methanol solvation for ***p*HBDI** and **26Me** are shown in Fig. S6. These surfaces were used to derive the S_1–S_0 energy splittings, $\Delta E(S_1–S_0)$, plotted in Fig. 3c & g of the manuscript. As discussed in the manuscript, the many low-frequency solvent degrees of freedom, together with the large number of local solvent configurations, make it difficult to ensure that each constrained φ_I geometry samples an equivalent solvent environment. Consequently, the absolute energies exhibit some configuration-dependent variation. However, the S_1–S_0 energy gap provides a more robust relative quantity and is consistent with the experimental dynamics and earlier AIMD trajectories by Martínez and co-workers described in the manuscript.

Our explicit solvation strategy involved taking each constrained φ_I geometry, solvating it using molecular-dynamics derived solvent configurations, and then reoptimising the cluster with MRSF-TDDFT. Future work could consider more integrated molecular dynamics approaches or averaging over several independently sampled solvent configurations, for example using parallel molecular dynamics configuration sampling.

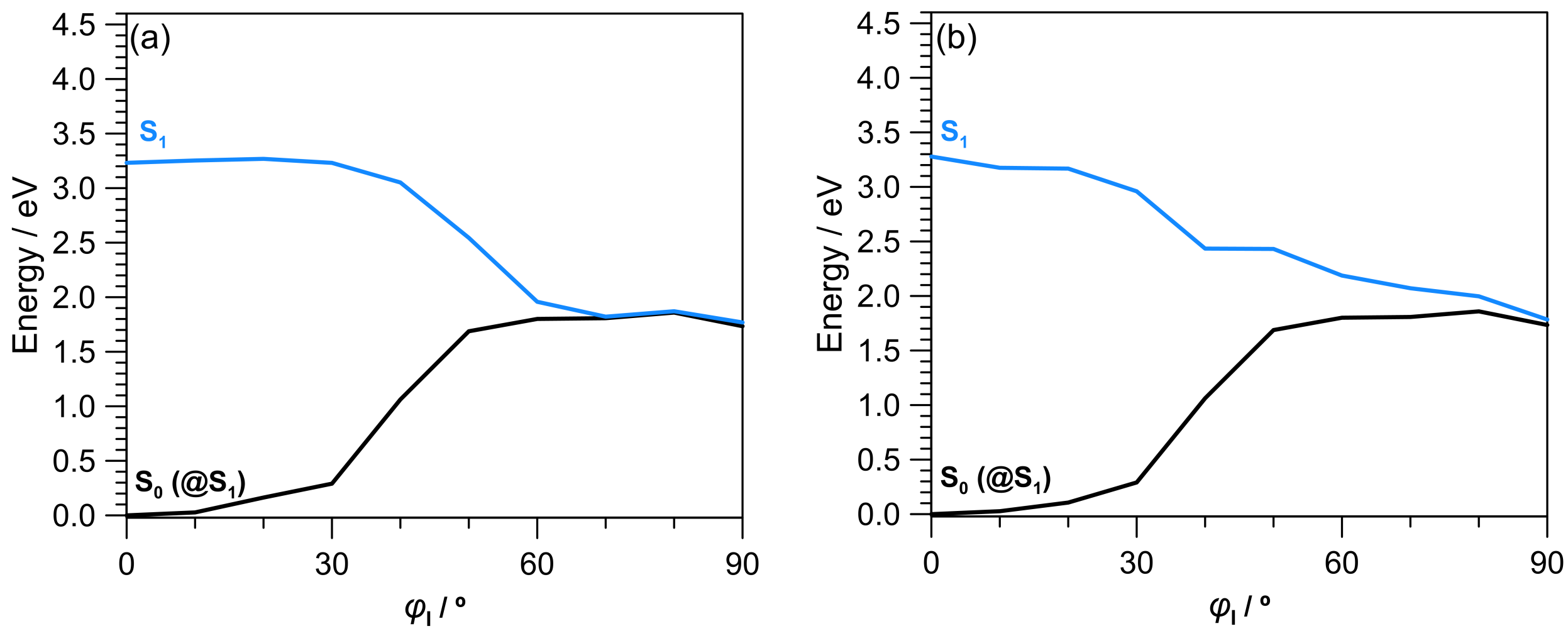

Fig. S6 Relaxed potential energy surfaces along φ_I using explicit methanol solvation: (a) pHBDI, and (b) 26Me.

9 Optimised geometries of critical points

	x	y	z
C	0.000732408700	-0.337621784800	1.073300993600
H	0.163569804700	-1.006550202600	1.926206412600
C	1.078783143400	0.342578025600	0.434560000300
C	2.093066885700	-0.424983888600	-0.121486265300
C	2.860754774200	1.465577382800	0.869855628400
N	1.612550892400	1.436246146100	1.184191727000
N	3.209390838200	0.422692902900	0.047246867200
C	3.820845443400	2.532913610900	1.263198984000
H	4.710169667600	2.125034531300	1.743862867000
H	4.161627361700	3.122267492200	0.410805870800
H	3.330037648900	3.202349041100	1.960947195100
O	2.145294689300	-1.541615073800	-0.681365042400
C	4.488740997000	0.106069513000	-0.508702169500
H	4.346210236600	-0.798258691000	-1.090624661300
H	4.858924995600	0.900547580300	-1.155147537300
H	5.236035201300	-0.086011100900	0.261016570100
C	-1.315845045800	-0.275356081200	0.713337862300
C	-1.707345069900	0.496165093000	-0.423705019900
C	-2.287477476500	-1.071854189700	1.392430707100
C	-2.991410932400	0.467751193100	-0.860526857300
H	-0.930718622700	1.059996526100	-0.901997204500
C	-3.567313303600	-1.117486635200	0.954617206600
H	-1.979219374800	-1.646496451500	2.249281570200
C	-3.916590692000	-0.353215820900	-0.186745317000
H	-3.328555041200	1.020096003900	-1.718760450500
H	-4.314362918300	-1.725029546500	1.438031486700
O	-5.129705310100	-0.384210147200	-0.674819615200
H	-5.706157266400	-0.978577323100	-0.186934610000

Table S1 pHBDI $S_{1,T}$.

	x	y	z
C	0.000732408700	-0.337621784800	1.073300993600
H	0.163569804700	-1.006550202600	1.926206412600
C	1.078783143400	0.342578025600	0.434560000300
C	2.093066885700	-0.424983888600	-0.121486265300
C	2.860754774200	1.465577382800	0.869855628400
N	1.612550892400	1.436246146100	1.184191727000
N	3.209390838200	0.422692902900	0.047246867200
C	3.820845443400	2.532913610900	1.263198984000
H	4.710169667600	2.125034531300	1.743862867000
H	4.161627361700	3.122267492200	0.410805870800
H	3.330037648900	3.202349041100	1.960947195100
O	2.145294689300	-1.541615073800	-0.681365042400
C	4.488740997000	0.106069513000	-0.508702169500
H	4.346210236600	-0.798258691000	-1.090624661300
H	4.858924995600	0.900547580300	-1.155147537300
H	5.236035201300	-0.086011100900	0.261016570100
C	-1.315845045800	-0.275356081200	0.713337862300
C	-1.707345069900	0.496165093000	-0.423705019900
C	-2.287477476500	-1.071854189700	1.392430707100
C	-2.991410932400	0.467751193100	-0.860526857300
H	-0.930718622700	1.059996526100	-0.901997204500
C	-3.567313303600	-1.117486635200	0.954617206600
H	-1.979219374800	-1.646496451500	2.249281570200
C	-3.916590692000	-0.353215820900	-0.186745317000
H	-3.328555041200	1.020096003900	-1.718760450500
H	-4.314362918300	-1.725029546500	1.438031486700
O	-5.129705310100	-0.384210147200	-0.674819615200
H	-5.706157266400	-0.978577323100	-0.186934610000

Table S2 pHBDI MECP.

	x	y	z
C	1.479477417500	0.988644826900	-0.999266042100
C	3.220488061300	0.109298257500	0.145784943000
C	1.102095495900	-0.003066581800	-0.086521380500
C	4.614837964100	-0.087546480300	0.618571903700
H	5.011237221700	0.801855565200	1.110002063200
H	4.623795692400	-0.902349323200	1.333640500100
H	5.298202544800	-0.333326994900	-0.194445585400
C	3.719261008800	1.952368846300	-1.507059332700
H	4.506670085600	1.429780439200	-2.046724019300
H	3.085751988700	2.473495405400	-2.215825234300
H	4.180490283200	2.678967553400	-0.840389002600
N	2.185395694600	-0.540210198500	0.586171931200
N	2.871239483500	1.041129421600	-0.796071993900
C	-0.233805396200	-0.506734437300	0.104612391800
H	-0.827054000100	-0.044000319100	0.884235471200
C	-0.884986225600	-1.510066962200	-0.562737717300
C	-0.302970026200	-2.219934401400	-1.691736101900
C	-2.227821317700	-1.871264562700	-0.126039441300
C	-1.032705620400	-3.216182542100	-2.285178486900
C	-2.900947894500	-2.872769737400	-0.753820804200
C	-2.309589581200	-3.548137219900	-1.831264820500
H	-0.621646523100	-3.750057105900	-3.127401217000
H	-3.889369449700	-3.169339915100	-0.449812833300
O	0.874534520400	1.651139094700	-1.859514613700
C	-2.901975672600	-1.175165591900	1.020161866500
H	-2.340654565300	-1.291105030100	1.943238427100
H	-3.008705705600	-0.110524706100	0.831438985000
H	-3.892002340600	-1.588431243100	1.176189833200
C	1.035735235000	-1.900248816300	-2.271703927300
H	1.044952129900	-0.878615236400	-2.640036587700
H	1.818475843000	-1.978452941200	-1.526132516900
H	1.247897092800	-2.577916925800	-3.093693257300
O	-3.026573750500	-4.507821235600	-2.394069984100
H	-2.550340682100	-4.914055554500	-3.121924579500

Table S3 26Me $S_{1,T}$.

	x	y	z
C	1.488785608000	0.966494017300	-1.148304312000
C	3.274220181700	0.277669616000	0.090467380600
C	1.313535741300	-0.276524771300	-0.578191226400
C	4.646941872400	0.269992373900	0.665475538300
H	4.874938812800	1.193506031300	1.198537599400
H	4.722911308400	-0.554677903000	1.365994174900
H	5.421460933400	0.142182218200	-0.092529125000
C	3.517445944300	2.403397948800	-1.249606861500
H	4.416418507000	2.159636634700	-1.815553658300
H	2.814971595000	2.921718360900	-1.893791018000
H	3.800241699300	3.063488631900	-0.429633022700
N	2.377697437700	-0.626710886400	0.293577699100
N	2.836488563100	1.233370026500	-0.789954250100
C	0.021507450500	-0.714219278400	-0.145004911700
H	-0.470850174800	-0.186173487000	0.669648709100
C	-0.748605964500	-1.726659210800	-0.638143793800
C	-0.289641313500	-2.591205404400	-1.720685400400
C	-2.125999833700	-1.826263232800	-0.166242046900
C	-1.207644429000	-3.406466203900	-2.319072593600
C	-2.996881344200	-2.642094170600	-0.811611086400
C	-2.543698293600	-3.411791116400	-1.898667559400
H	-0.909435511600	-4.061943235400	-3.120508858700
H	-4.029577715600	-2.727132187400	-0.525899638200
O	0.789768383300	1.748015006500	-1.836639951600
C	-2.641862217800	-1.032438315300	0.999261380100
H	-2.024569921900	-1.171731975800	1.879869288700
H	-2.665658260400	0.028263095600	0.771389751300
H	-3.651335710600	-1.346268395200	1.237857129800
C	1.122573062500	-2.685149788000	-2.192775551300
H	1.356656329800	-1.810578028100	-2.784690081600
H	1.821774852900	-2.665837798000	-1.370478294100
H	1.240957069400	-3.586229345100	-2.788764495400
O	-3.440631734600	-4.157779527600	-2.487522641600
H	-3.068791586600	-4.657771891300	-3.219820531800

Table S4 26Me MECP.

Notes and references

1 C. R. Hall, J. Conyard, I. A. Heisler, G. Jones, J. Frost, W. R. Browne, B. L. Feringa and S. R. Meech, *J. Am. Chem. Soc.*, 2017, **139**, 7408–7414.

2 J. J. Snellenburg, S. P. Laptenok, R. Seger, K. M. Mullen and I. H. M. van Stokkum, *J. Stat. Soft.*, 2012, **49**, 1–22.

3 G. M. Greetham, P. Burgos, Q. Cao, I. P. Clark, P. S. Codd, R. C. Farrow, M. W. George, M. Kogimtzis, P. Matousek, A. W. Parker, M. R. Pollard, D. A. Robinson, Z.-J. Xin and M. Towrie, *Appl. Spec.*, 2010, **64**, 1311–1319.

4 J. N. Bull, M. H. Stockett, P. Chakraborty, E. K. Ashworth, A. Fatima, V. J. Esposito, G. M. Greetham, P. Malakar and S. R. Meech, *J. Phys. Chem. B*, 2025, **129**, 13267–13276.

5 W. Park, K. Komarov, S. Lee and C. H. Choi, *J. Phys. Chem. Lett.*, 2023, **14**, 8896–8908.

6 S. Lee, W. Park and C. H. Choi, *Acc. Chem. Res.*, 2025, **58**, 208–217.

7 G. M. J. Barca, C. Bertoni, L. Carrington, D. Datta, N. De Silva, J. E. Deustua, D. G. Fedorov, J. R. Gour, A. O. Gunina, E. Guidez, T. Harville, S. Irle, J. Ivanic, K. Kowalski, S. S. Leang, H. Li, W. Li, J. J. Lutz, I. Magoulas, J. Mato, V. Mironov, H. Nakata, B. Q. Pham, P. Piecuch, D. Poole, S. R. Pruitt, A. P. Rendell, L. B. Roskop, K. Ruedenberg, T. Sattasathuchana, M. W. Schmidt, J. Shen, L. Slipchenko, M. Sosonkina, V. Sundriyal, A. Tiwari, J. L. Galvez Vallejo, B. Westheimer, M. Włoch, P. Xu, F. Zahariev and M. S. Gordon, *J. Chem. Phys.*, 2020, **152**, 154102.

8 V. Mironov, K. Komarov, J. Li, I. Gerasimov, H. Nakata, M. Mazaherifar, K. Ishimura, W. Park, A. Lashkaripour, M. Oh, M. Huix-Rotllant, S. Lee and C. H. Choi, *J. Chem. Theo. Comput.*, 2024, **20**, 9464–9477.

9 S. Lee, S. Shostak, M. Filatov and C. H. Choi, *J. Phys. Chem. A*, 2019, **123**, 6455–6462.

10 N. T. Maitra, F. Zhang, R. J. Cave and K. Burke, *J. Chem. Phys.*, 2004, **120**, 5932–5937.

11 B. G. Levine, C. Ko, J. Quenneville and T. J. Martinez, *Mol. Phys.*, 2006, **104**, 1039–1051.

12 A. D. Becke, *J. Chem. Phys.*, 1993, **98**, 1372–1377.

13 M. M. Francl, W. J. Pietro, W. J. Hehre, J. S. Binkley, M. S. Gordon, D. J. DeFrees and J. A. Pople, *J. Chem. Phys.*, 1982, **77**, 3654–3665.

14 M. Huix-Rotllant, W. Park, M. Mazaherifar and C. H. Choi, *Theor. Chem. Acc.*, 2025, **144**, 41.

15 A. V. Marenich, C. J. Cramer and D. G. Truhlar, *J. Phys. Chem. B*, 2009, **113**, 6378–6396.

16 C. Bannwarth, S. Ehlert and S. Grimme, *J. Chem. Theo. Comput.*, 2019, **15**, 1652–1671.

17 A. Fatima, E. K. Ashworth, I. Chambrier, A. N. Cammidge, G. Bressan, S. R. Meech and J. N. Bull, *Phys. Chem. Chem. Phys.*, 2025, **27**, 9407–9416.

18 G. Bressan, K. M. Siddiqui, E. K. Ashworth, P. Chakraborty, D. Banerjee, E. M. Braun, S. R. Meech and J. N. Bull, *Phys. Chem. Chem. Phys.*, 2026, **28**, 1260–1268.

19 F. Neese, *WIREs Comput. Mol. Sci.*, 2011, **2**, 73–78.

20 V. Barone, *J. Chem. Phys.*, 2005, **122**, 014108.

21 V. Barone, P. Cimino and E. Stendardo, *J. Chem. Theory Comput.*, 2008, **4**, 751–764.

22 M. J. Frisch, G. W. Trucks, H. B. Schlegel, G. E. Scuseria, M. A. Robb, J. R. Cheeseman, G. Scalmani, V. Barone, G. A. Petersson, H. Nakatsuji, X. Li, M. Caricato, A. V. Marenich, J. Bloino, B. G. Janesko, R. Gomperts, B. Mennucci, H. P. Hratchian, J. V. Ortiz, A. F. Izmaylov, J. L. Sonnenberg, D. Williams-Young, F. Ding, F. Lipparini, F. Egidi, J. Goings, B. Peng, A. Petrone, T. Henderson, D. Ranasinghe, V. G. Zakrzewski, J. Gao, N. Rega, G. Zheng, W. Liang, M. Hada, M. Ehara, K. Toyota, R. Fukuda, J. Hasegawa, M. Ishida, T. Nakajima, Y. Honda, O. Kitao, H. Nakai, T. Vreven, K. Throssell, J. A. Montgomery, Jr., J. E. Peralta, F. Ogliaro, M. J. Bearpark, J. J. Heyd, E. N. Brothers, K. N. Kudin, V. N. Staroverov, T. A. Keith, R. Kobayashi, J. Normand, K. Raghavachari, A. P. Rendell, J. C. Burant, S. S. Iyengar, J. Tomasi, M. Cossi, J. M. Millam, M. Klene, C. Adamo, R. Cammi, J. W. Ochterski, R. L. Martin, K. Morokuma, O. Farkas, J. B. Foresman and D. J. Fox, *Gaussian 16 Revision B.01*, 2016, Gaussian Inc. Wallingford CT.

23 V. Barone, M. Biczysko and J. Bloino, *Phys. Chem. Chem. Phys.*, 2014, **16**, 1759–1787.

24 C. Puzzarini, M. Biczysko and V. Barone, *J. Chem. Theor. Comp.*, 2010, **6**, 828–838.

25 T. E. Douglas-Walker, E. K. Ashworth, M. H. Stockett, F. C. Daly, I. Chambrier, V. J. Esposito, M. Gerlach, A. Zheng, J. Palotás, A. N. Cammidge, E. K. Campbell, S. Brünken and J. N. Bull, *ACS Earth Space Chem.*, 2024, **9**, 134–145.

26 M. H. Stockett, V. J. Esposito, E. K. Ashworth, U. Jacovella and J. N. Bull, *ACS Earth Space Chem.*, 2025, **9**, 382–393.

27 M. Ceriotti, G. Bussi and M. Parrinello, *Phys. Rev. Lett.*, 2009, **102**, 020601.

28 M. Ceriotti, G. Bussi and M. Parrinello, *J. Chem. Theor. Comput.*, 2010, **6**, 1170–1180.

29 D. Hollas, J. Suchan, M. Oncčák, O. Svoboda and P. Slavíček, *ABIN: Source code available at https://github.com/PHOTOX/ABIN*, 2021.

30 GLE4MD Project, *GLE4MD*, `http://gle4md.org/`, 2025, accessed 2 September 2025.

31 A. Prlj, D. Hollas and B. F. E. Curchod, *J. Phys. Chem. A*, 2023, **127**, 7400–7409.

32 J. C. Tully, *J. Chem. Phys.*, 1990, **93**, 1061–1071.

33 S. Lee, E. E. Kim, H. Nakata, S. Lee and C. H. Choi, *J. Chem. Phys.*, 2019, **150**, 184111.

34 S. Lee, Y. Horbatenko, M. Filatov and C. H. Choi, *J. Phys. Chem. Lett.*, 2021, **12**, 4722–4728.

35 R. Mitrić, U. Werner and V. Bonačić-Koutecký, *J. Chem. Phys.*, 2008, **129**, 164118.

36 W. Park, J. Oh, J. Kim, S. Lee, J. H. Kim, M. Huix-Rotllant, D. Kim and C. H. Choi, *J. Phys. Chem. Lett.*, 2024, **15**, 11468–11475.

37 B. H. Bransden and C. J. Joachain, *Quantum mechanics*, Pearson/Prentice Hall, Harlow, 2nd edn., 2007.

38 P.-O. Löwdin, *Phys. Rev.*, 1955, **97**, 1474–1489.
